# Global metabolite profiling analysis of lipotoxicity in HER2/neu-positive breast cancer cells

**DOI:** 10.18632/oncotarget.25500

**Published:** 2018-06-05

**Authors:** Jan Baumann, Mostafa Kokabee, Jason Wong, Rakshika Balasubramaniyam, Yan Sun, Douglas S. Conklin

**Affiliations:** ^1^ Cancer Research Center, Department of Biomedical Sciences, State University of New York, University at Albany, Rensselaer, NY 12144, USA

**Keywords:** HER2/neu, metabolism, palmitate, high fat diet, nitrogen metabolism

## Abstract

Recent work has shown that HER2/neu-positive breast cancer cells rely on a unique Warburg-like metabolism for survival and aggressive behavior. These cells are dependent on fatty acid (FA) synthesis, show markedly increased levels of stored fats and disruption of the synthetic process results in apoptosis. In this study, we used global metabolite profiling and a multi-omics network analysis approach to model the metabolic changes in this physiology under palmitate-supplemented growth conditions to gain insights into the molecular mechanism and its relevance to disease prevention and treatment. Computational analyses were used to define pathway enrichment based on the dataset of significantly altered metabolites and to integrate metabolomics and transcriptomics data in a multi-omics network analysis. Network-predicted changes and functional relationships were tested with cell assays *in vitro*. Palmitate-supplemented growth conditions induce distinct metabolic alterations. Growth of HER2-normal MCF7 cells is unaffected under these conditions whereas HER2/neu-positive cells display unchanged neutral lipid content, AMPK activation, inhibition of fatty acid synthesis and significantly altered glutamine, glucose and serine/glycine metabolism. The predominant upregulated lipid species is the novel bioactive lipid N-palmitoylglycine, which is non-toxic to these cells. Limiting the availability of glutamine significantly ameliorates the lipotoxic effects of palmitate, reduces CHOP and XBP1(s) induction and restores the expression levels of HER2 and HER3. The study shows that HER2/neu-positive breast cancer cells change their metabolic phenotype in the presence of palmitate. Palmitate induces AMPK activation and inhibition of fatty acid synthesis that feeds back into glycolysis as well as anaplerotic glutamine metabolism.

## INTRODUCTION

Amplification of the ERBB2 (HER2) oncogene is one of the most clinically relevant genetic changes in breast cancer. It occurs in about 20% of all breast cancer cases [1] and is associated with resistance to chemotherapy, increased recurrence and worse prognosis [2]. However, several studies have shown that a number of genes are frequently co-overexpressed or co-amplified along with HER2 [3–5]. Functional genomics studies revealed that several of these co-overexpressed genes are required for HER2/neu-positive breast cancer cell survival, many of which are known to be involved in fat metabolism and adipogenesis [6]. PPARγ binding protein (PBP) and the nuclear receptor NR1D1 are two pro-adipogenic transcriptional regulators that cooperatively change the metabolism of HER2/neu-positive breast cancer cells, inducing a unique, Warburg-like metabolism that is primed towards fat production by increasing the expression of pro-lipogenic enzymes [6].

Overexpression of HER2 alone has previously been shown to have pro-lipogenic effects, i.e. by increasing the expression of acetyl-CoA carboxylase alpha (ACACA/ACC) and fatty acid synthase (FASN) at the translational level [7]. These pro-lipogenic alterations in HER2/neu-positive breast cancer cells are congruent with the observation that these cells possess higher levels of stored triacylglycerides (TAGs) as well as higher levels of saturated fatty acids compared to other cell types [6, 8]. This lipogenic phenotype and altered metabolism of HER2/neu-positive breast cancer cells has been reviewed recently [9–12]. The genetic alterations in these cells allow for the constant production of fatty acids as a means to regenerate reducing equivalents for glycolysis through the concerted action of malic enzyme (ME1) and malate dehydrogenase (MDH1), while PBP, NRD1 and PPARγ orchestrate the sequestration of fatty acids in neutral lipids to avoid lipotoxicity [6, 8] [13].

The lipogenic metabolism of HER2/neu-positive breast cancer cells leaves them especially sensitive to physiological concentrations of exogenous saturated fatty acids, such as palmitate. Palmitate supplementation induces cell death in HER2/neu-positive breast cancer cells but not other breast cancer cells or normal human mammary epithelial cells (HMECs) [8, 14]. Palmitate supplementation significantly reduces the expression levels of HER2 and HER3, sensitizes cells to trastuzumab and induces ER-stress and CHOP-dependent apoptosis *in vitro* [14]. That this stress is relevant to tumor biology is evidenced by the increased expression of ER stress response genes in HER2/neu-positive breast tumors [14]. The underlying cause of this lipotoxicity is most likely due to a combination of genetic alterations that exist in HER2/neu-positive breast cancer cells, considering that the HER2 amplicon has been shown to comprise several genes in a large section on chromosome 17 [3, 4, 15–19] and other genes have been shown to be co-overexpressed and required for breast cancer cell survival [6]. This sensitivity to lipotoxicity may have consequences in patient populations in light of a recent epidemiological study. The investigators followed 337,327 women for 11.5 years and evaluated fat intake as a predictor of breast cancer development and found that a diet high in saturated fatty acids was positively associated with the development of HER2/neu-negative disease, but not HER2/neu-positive disease [20]. In this study, we have used global metabolite profiling and a multi-omics network analysis approach to identify the metabolic changes that result from stressing the Warburg-like physiology of HER2/neu-positive breast cancer cells with exogenous palmitate. The work provides insights into the molecular basis of the lipotoxic phenotype and its relevance to disease prevention and treatment.

## RESULTS

### Supplementation of culture media with saturated fatty acids induces distinct responses in breast cancer cells

**Figure 1:**
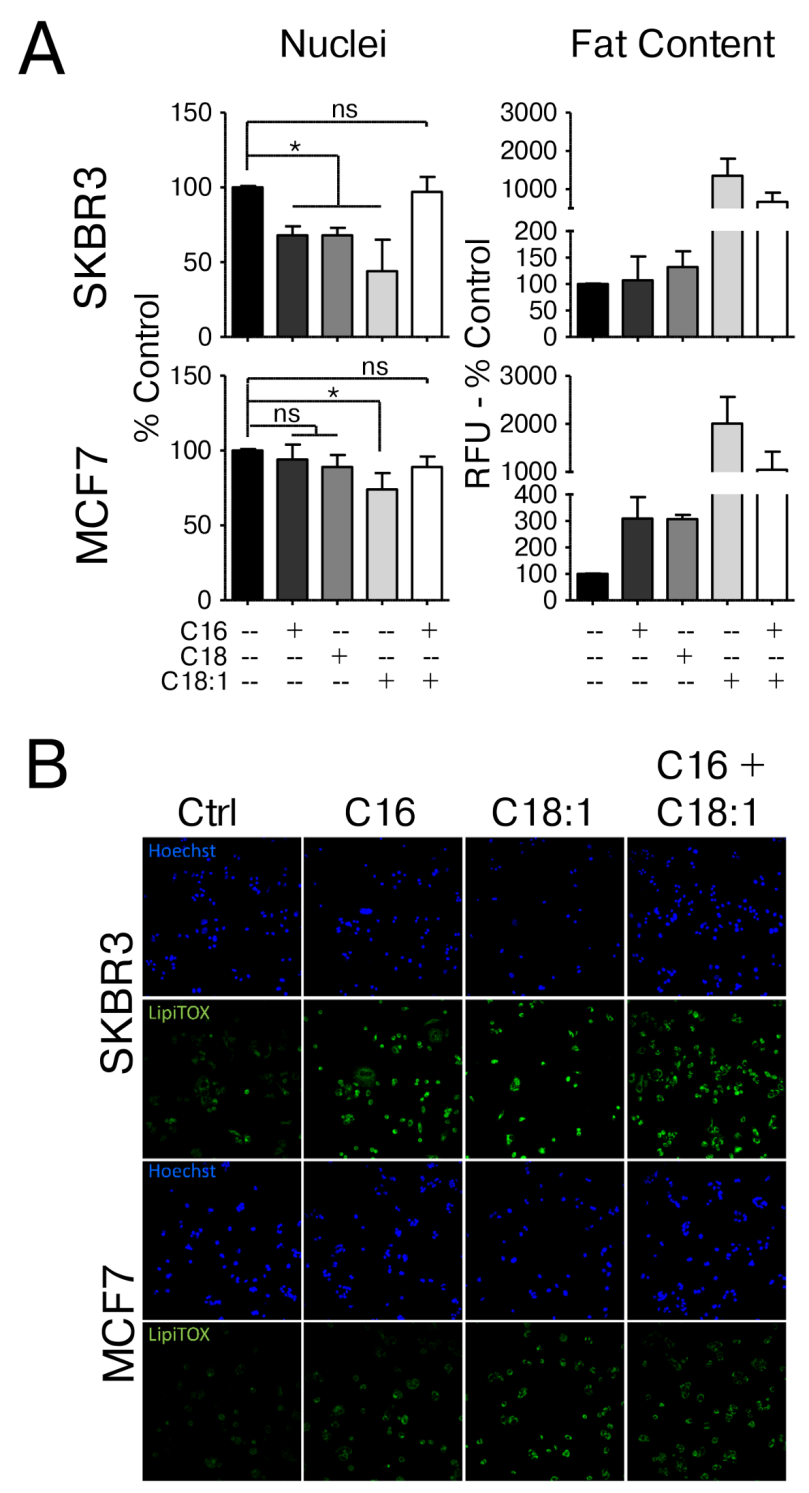
HER2/neu-positive SKBR3 and HER2-normal MCF7 cells differ in their response to saturated fatty acid supplementation **(A)** HER2/neu-positive SKBR3 and HER2-normal MCF7 breast cancer cells were treated with 250μM palmitate, stearate, oleate, palmitate + oleate or vehicle for 24 h. Cells were fixed and neutral lipids were stained with BODIPY 493/503. Nuclei were stained with Hoechst 33342. BODIPY fluorescence and nuclei were imaged and quantified using the INCell Analyzer 2200 and INCell Investigator software. Fluorescence intensity per cell is proportional to the neutral lipid content in the cell. **(B)** HER2/neu-positive SKBR3 and HER2-normal MCF7 breast cancer cells were treated with 250μM palmitate, oleate, palmitate + oleate or vehicle for 24 h. Cells were fixed and neutral lipids were stained with LipidTOX DeepRed. Nuclei were stained with Hoechst 33342 and fluorescent images were taken using the INCell Analyzer 2200. Statistical analysis was carried out in Graphpad Prism. Data are presented as mean + SD. Between 3000 and 7000 cells were analyzed per experiment. ^*^ = p < 0.05, one-way ANOVA with Bonferroni post-test, n = 3.

HER2/neu-positive breast cancer cells contain high levels of endogenous saturated fatty acids and neutral lipids and generally exhibit a pro-lipogenic phenotype. Our previous studies have established that BT474 (luminal B; ER^+^, HER2^+^), MDA-MB-361 (luminal B; ER^+^, HER2^+^), SKBR3 (HER2 enriched; ER^-^, HER2^+^) but not MCF-7 (luminal A; ER^-^, HER2^wt^) or human mammary epithelial cells exhibit this Warburg-like physiology which relies on active fatty acid synthesis for survival and aggressive behavior [6, 8-10, 14, 21]. Additionally, molecular profiling experiments from this work have shown that the MCF7 cell line (HER2-normal) and the SKBR3 cell line (HER2/neu-positive) are representative lines to investigate the differential effects of fatty acids as a model of increased dietary fat intake. The use of MCF7 cells as a control is preferable since they can be grown in the same culture medium as SKBR3 cells and previous studies have shown that the response to exogenous fatty acids in MCF7 cells is comparable to that of non-tumorigenic MCF10A mammary epithelial cells or normal human mammary epithelial cells (HMECs) [8, 22]. We cultured MCF7 and SKBR3 cells in the presence of either 250 μM palmitate (C16), stearate (C18), oleate (C18:1) or palmitate and oleate in combination (250 μM and 150 μM, respectively) and monitored cell count as well as levels of intracellular neutral fat stores compared to vehicle control. Supplementing the growth media with the saturated fatty acids palmitate and stearate significantly reduces the number of SKBR3 cells, but not MCF7 cells, indicating the induction of distinct responses to saturated fat in the two cell lines. These effects are mediated by the effects of palmitate on cellular physiology and not as effects on cellular integrity which are not seen at concentrations in this range [14]. This distinction is further evidenced through observed changes in lipid content. While SKBR3 cells show higher basal levels of stored neutral fats that do not change with palmitate or stearate treatment, MCF7 cells display low basal neutral fat content which increases significantly upon saturated fatty acid exposure (Figure 1A, 1B and Supplementary Figure 1). In SKBR3 cells, palmitate has been shown to induce a partial ER-stress response and CHOP-dependent apoptosis [14]. Supplementation with the mono-unsaturated fatty acid oleate, however, significantly reduces the cell number in both lines. Interestingly, simultaneous supplementation of oleate and palmitate completely abrogates the observed toxicity of palmitate supplementation in the HER2/neu-positive SKBR3 breast cancer cells, even though the total amount of supplemented FAs exceeds that of palmitate alone. Under these conditions, neutral lipid stores are significantly increased from basal levels in SKBR3 cells without significant effects on cell numbers, indicating that general defects in the TAG synthesis pathways are unlikely to be the cause for the saturated fatty acid-induced cytotoxicity, however defects in the processing of each individual FA may still exist (Figure 1A, 1B and Supplementary Figure 1). These results with SKBR3 are typical of HER2/neu-positive breast cancer cells which undergo distinct physiological changes upon exposure to saturated fat and sublethal concentrations. MCF10A cells transduced with either wild-type or activated ERBB2/HER2/neu do not display this sensitivity [14] indicating that genetic changes other than amplification of the oncogene are responsible [8, 14].

### Global metabolite profiling identifies specific pathways regulated by palmitate supplementation

**Figure 2:**
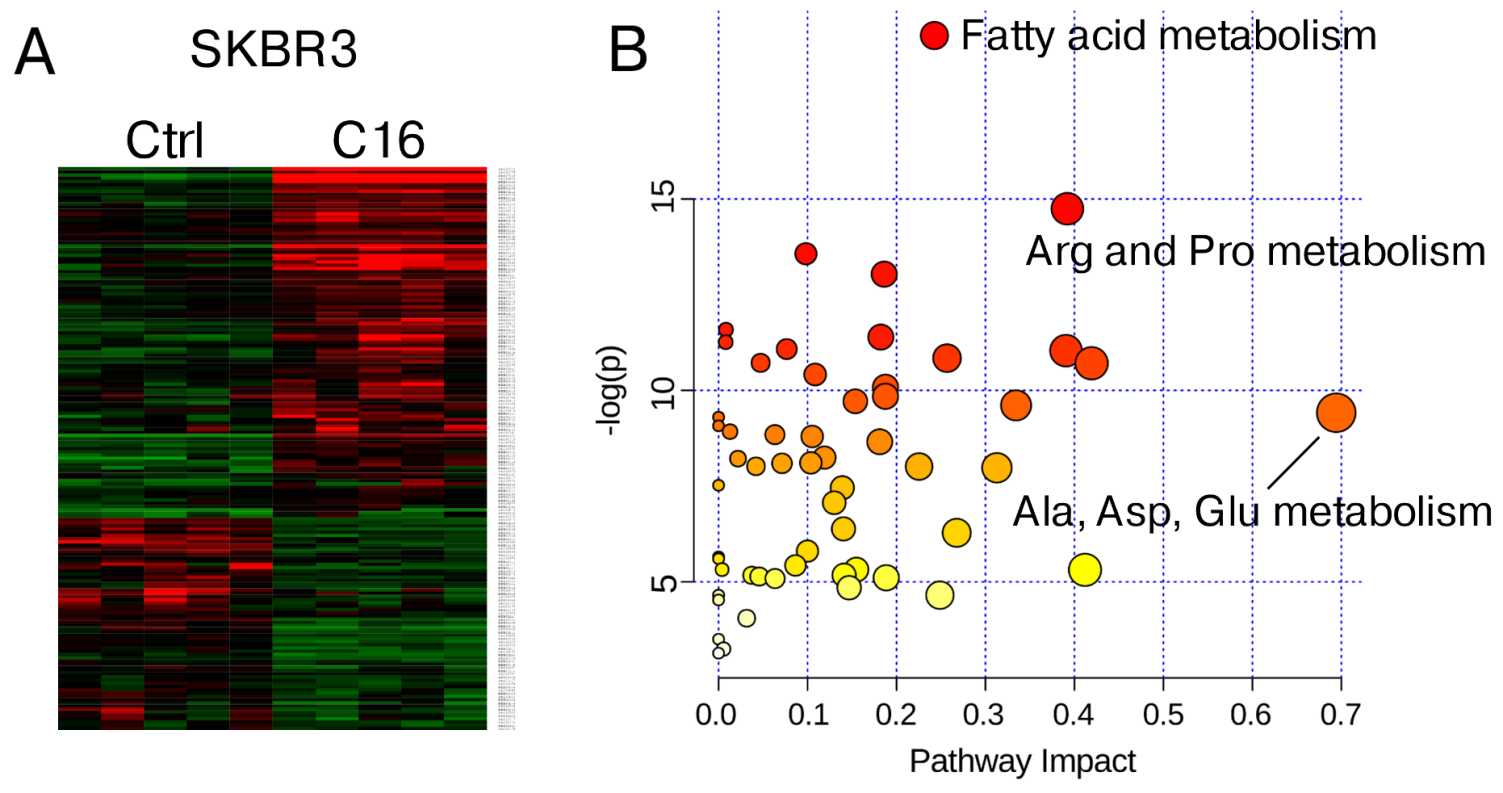
Metabolite profiling of palmitate-treated HER2/neu-positive SKBR3 breast cancer cells HER2/neu-positive SKBR3 were treated with 250μM palmitate or vehicle for 24 h in quintuplicate. Cells were harvested by trypsinization and subjected global metabolite profiling analysis by Metabolon Inc. **(A)** Heatmap showing significantly altered metabolites between vehicle and palmitate treated samples (n = 5). **(B)** Metabolite pathway enrichment was performed using the web resource at www.metaboanalyst.ca. Metabolites were mapped to database identifiers and subsequently subjected to pathway enrichment analysis using the GlobalTest algorithm. Node importance for pathway topology analysis was determined using the “relative betweenness centrality” metric.

While MCF7 cells appear to effectively sequester excess palmitate in neutral lipids, the effects induced by palmitate supplementation in HER2/neu-positive SKBR3 cells, aside from causing cell death, are less obvious. We performed global metabolite profiling of SKBR3 cells subjected to either sublethal treatment with 250 μM palmitate or vehicle control for 24 h and identified 168 metabolites with significantly altered concentrations at p=0.05. Using a less stringent 90% confidence interval, an additional 44 compounds change consistently among the samples (Table 1). A heatmap representation of the data, generated by hierarchical clustering, shows clear separation of the samples by growth condition (Figure 2A). To evaluate whether specific pathways are altered by palmitate supplementation we used the bioinformatics resource (www.metaboanalyst.ca). Metabolites were mapped to database identifiers and subsequently subjected to pathway enrichment analysis. The list of metabolites can be found in Supplementary Table 1. Based on the fact that many lipid species, e.g. mono- and diacylglycerols, as well as phospholipid precursors, do not currently have an assigned KEGG or HMDB database identifier, these species are not represented in our computational analysis. Figure 2B shows the results of the pathway topology analysis, which assigns a pathway impact score and a p-value, based on metabolite concentrations and its position in the metabolic network [23, 24]. Three nodes show significant enrichment based on p-value (fat metabolism) and pathway impact (Ala, Asp, Glu metabolism) or both (Arg and Pro metabolism). The full list of results of the pathway enrichment analysis can be found in Supplementary Table 2. While the enrichment of metabolites associated with fat metabolism is expected, it is surprising that two pathways that are connected through glutamine/glutamate metabolism were significantly enriched under palmitate supplemented growth conditions.

**Table 1:** Summary of the global metabolite profiling analysis

	Palmitate vs. Control		
	Metabolites identified	up	down
p ≤ 0:05	168	115	53
0:05 < p < 0:1	44	26	18

Following normalization to Bradford protein concentration, median scaling, imputation of missing values, if any, with the minimum observed value for each compound and log transformation of median scaled data, Welch’s two-sample *t*-test was used to identify biochemicals that differed significantly between experimental groups.

### Palmitate supplementation results in AMPK activation and inhibition of ACACA

**Figure 3:**
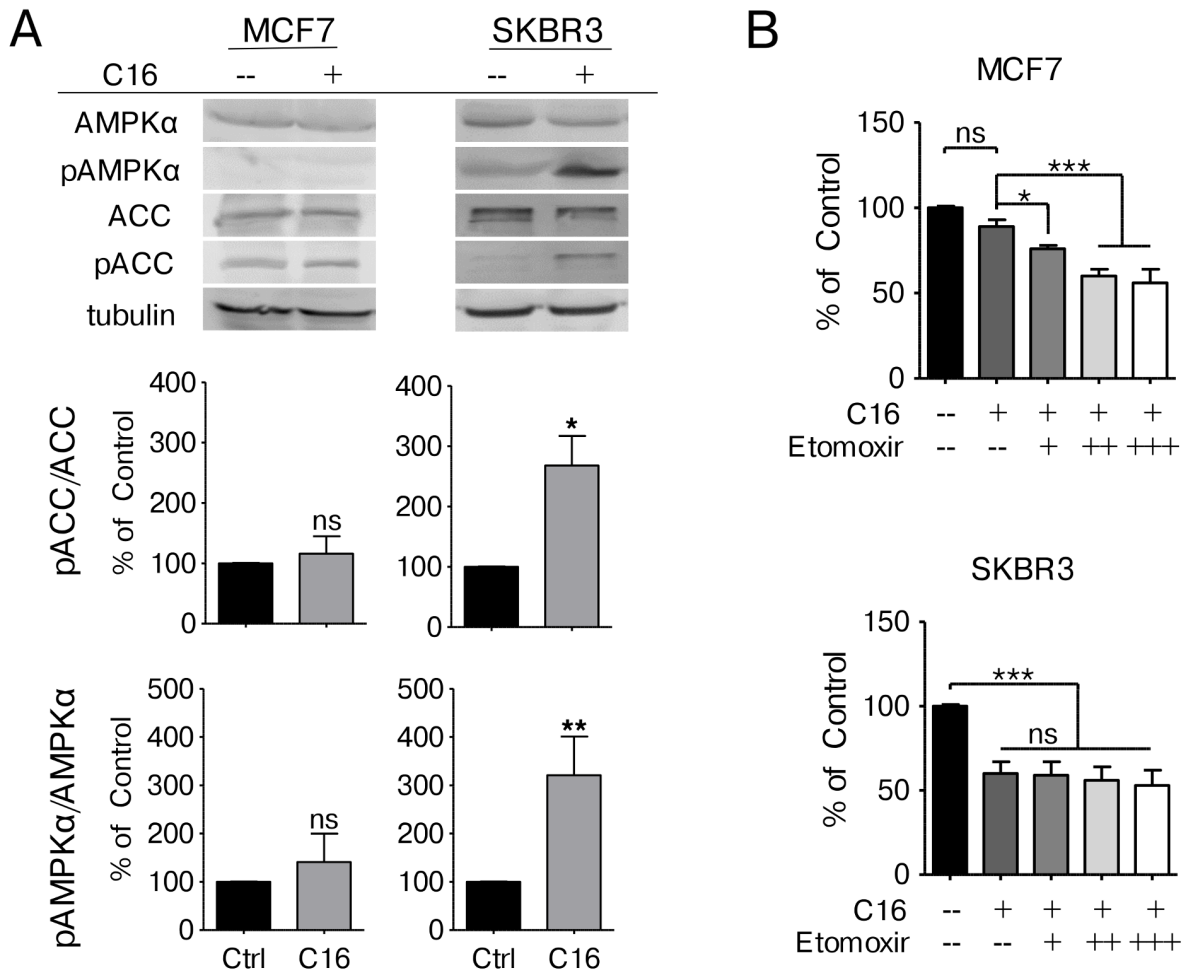
Exogenous palmitate inhibits FA synthesis in HER2/neu-positive SKBR3 breast cancer cells **(A)** MCF7 and SKBR3 cells were treated with 250 μM palmitate or vehicle for 24 h. Total protein was harvested and subjected to western blot analysis to evaluate the protein expression and phosphorylation status of ACC, phospho-ACC (pACC, Ser79), AMPKα and phospho-AMPKα (pAMPKα, Thr172). Each experiment was performed in triplicate, representative images are shown. Signal was quantified using ImageJ software. **(B)** MCF7 and SKBR3 cells were treated with 250 μM palmitate or vehicle for 24 h, alone or in combination with the CPT-1 inhibitor Etomoxir (+ = 100 μM). After fixation, nuclei were stained with Hoechst 33342 and quantified using the INCell Analyzer 2200 and INCell Investigator software. Statistical analysis was carried out in Graphpad Prism. Data were normalized to control conditions and are presented as mean ± SD. ^*^= p ≤ 0.05, ^**^ = p ≤ 0.01, ^***^ = p ≤ 0.001, Student’s *t*-test (A), one-way ANOVA with Bonferroni post-test (B), n = 3.

To further elucidate how fat metabolism is impacted by palmitate supplementation we analyzed the expression levels and activation status of acetyl-CoA carboxylase α (ACACA/ACC), the rate limiting step of fatty acid synthesis, by immunoblot. MCF7 cells show only low levels of ACC expression with equivalent levels of inhibitory phosphorylation at serine 79. Neither ACC protein levels nor the phosphorylation status change with palmitate supplementation in MCF7 cells. SKBR3 cells however display high basal expression of ACC with very low serine 79 phosphorylation that increases significantly with palmitate supplementation (Figure 3A). Serine 79 on ACC is a known target of the energy sensor AMP-activated protein kinase (AMPK). AMPK is activated under low energy conditions through phosphorylation of threonine 172 in the alpha-subunit [25]. Palmitate supplementation results in a significant increase of phosphorylated AMPKα in the HER2/neu-positive SKBR3 cells whereas no change is detected in MCF7 cells (Figure 3A), corroborating the results obtained for ACC phosphorylation. AMPK activation has also been linked to increased β-oxidation [26]. To test whether changes in β-oxidation activity are involved in the lipotoxicity phenotype observed in SKBR3 cells we cultured cells in palmitate-supplemented media in the presence of the carnitine-palmitoyl transferase (CPT1) inhibitor etomoxir. CPT1 catalyzes the translocation of fatty acids from the cytosol into the mitochondria and represents the rate limiting step in palmitate oxidation [27, 28]. Increasing doses of etomoxir significantly inhibit the ability of MCF7 cells to deal with palmitate supplemented growth media (Figure 3B). In SKBR3 cells however, etomoxir treatment has no effect, indicating that the translocation of palmitate into the mitochondria for beta-oxidation is not involved in the lipotoxicity response (Figure 3B). These data indicate that palmitate supplementation inhibits fatty acid synthesis in HER2/neu-positive SKBR3 breast cancer cells through the activation of AMPK.

### Palmitate supplementation induces alterations in arginine and polyamine metabolism

**Figure 4:**
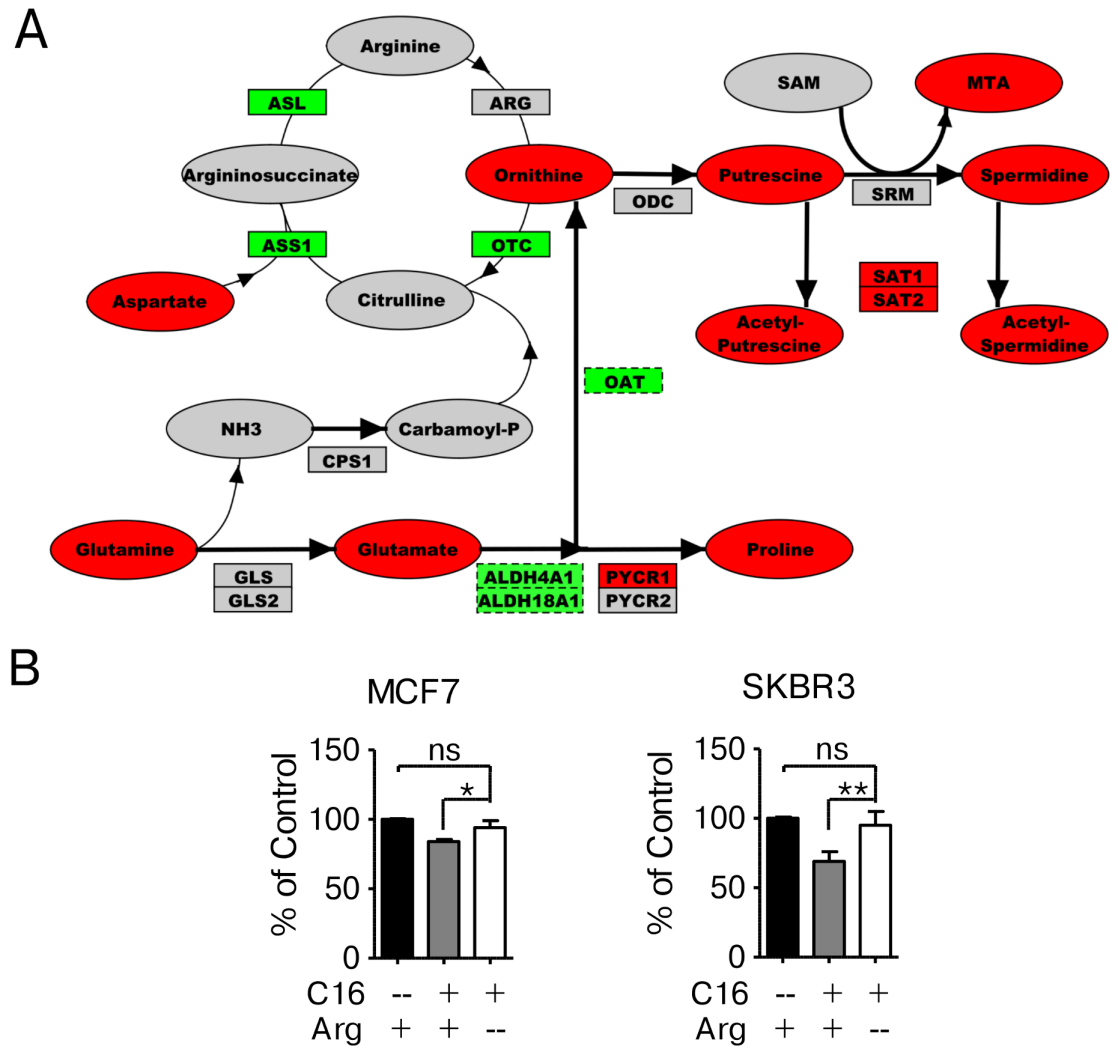
Network-overlay of metabolomic and transcriptomic data indicates alterations in arginine and polyamine metabolism **(A)** Detailed section of a network-overlay of metabolomic and transcriptomic data of SKBR3 cells after 24 h treatment with 250 μM palmitate. Arginine and proline metabolism are directly connected to glutamine metabolism as well as to polyamine biosynthesis. Networks were created in Metscape, detailed section were ported into PathVisio and edited for clarity. Oval nodes indicate metabolites, boxes indicate genes. Red fill color denotes upregulation, green downregulation compared to control conditions. Grey nodes are part of the network but were not identified or did not change in abundance/expression. Dashed outline of a node indicates p ≤ 0.1, whereas solid outlines indicate p ≤ 0.05. **(B)** MCF7 and SKBR3 cells were treated with 250 μM palmitate for 24 h in full growth medium or drop-out medium without arginine. After fixation, nuclei were stained with Hoechst 33342 and quantified using the INCell Analyzer 2200 and INCell Investigator software. The data were normalized to control conditions and are presented as mean ± SD. ^*^ = p ≤ 0.05, ^**^ = p ≤ 0.01, one-way ANOVA with Bonferroni post-test. N = 3.

To better understand the significance of the metabolic changes induced in SKBR3 cells by palmitate supplementation we performed a network-based analysis. Using the Cytoscape plugin Metscape we created a metabolomic-genomic network overlay using the global metabolic profiling data described in this study and transcriptional profiling data of palmitate treated SKBR3 cells [14]. By mapping gene and metabolite identifiers to reactions, regulatory differences in gene expression are correlated with metabolite concentration changes to visualize which pathways display consistent changes on the transcript and the metabolite level [29–31]. Using this global network, we evaluated the contributions of the enriched pathways identified in Figure 2B to palmitate-induced lipotoxicity. Figure 4A shows a section of this gene-metabolite network encompassing arginine, proline and associated metabolites observed in HER2/neu-positive SKBR3 cells. While arginine was not detected in our initial metabolic screen, several associated compounds are significantly altered in SKBR3, BT474 and MCF7 cell lines treated with palmitate suggesting that this is likely a general response to palmitate (Supplementary Table 3). These compounds include upstream metabolites like glutamine, glutamate and aspartate as well as downstream metabolites in the polyamine pathway, putrescine and spermidine. High levels of methylthioadenosine indicate an upregulation of polyamine biosynthesis as this metabolite is specific for this pathway. Increased expression of spermidine/spermine-N1-acetyltransferases (SAT1/SAT2), which acetylate spermine/putrescine using acetyl-CoA as a cofactor, not only links this pathway to fatty acid synthesis but may indicate increased excretion of acetylated polyamines to eliminate excess carbon and nitrogen [32]. Arginine is a non-essential amino acid and can be synthesized in the cells from glutamine. Limiting the availability of arginine in the growth medium of cells is expected to induce the enzymes required for biosynthesis while reducing the activity of consuming reactions. Additionally, this will induce the rate of nitrogen/ammonia sequestration, which is generated through glutamine catabolism. Growing the cells in arginine-deficient, palmitate-supplemented growth medium significantly reduces the growth-inhibitory effects compared to control conditions (Figure 4B).

### Palmitate supplementation induces alterations in glutamine, glucose and serine metabolism

**Figure 5:**
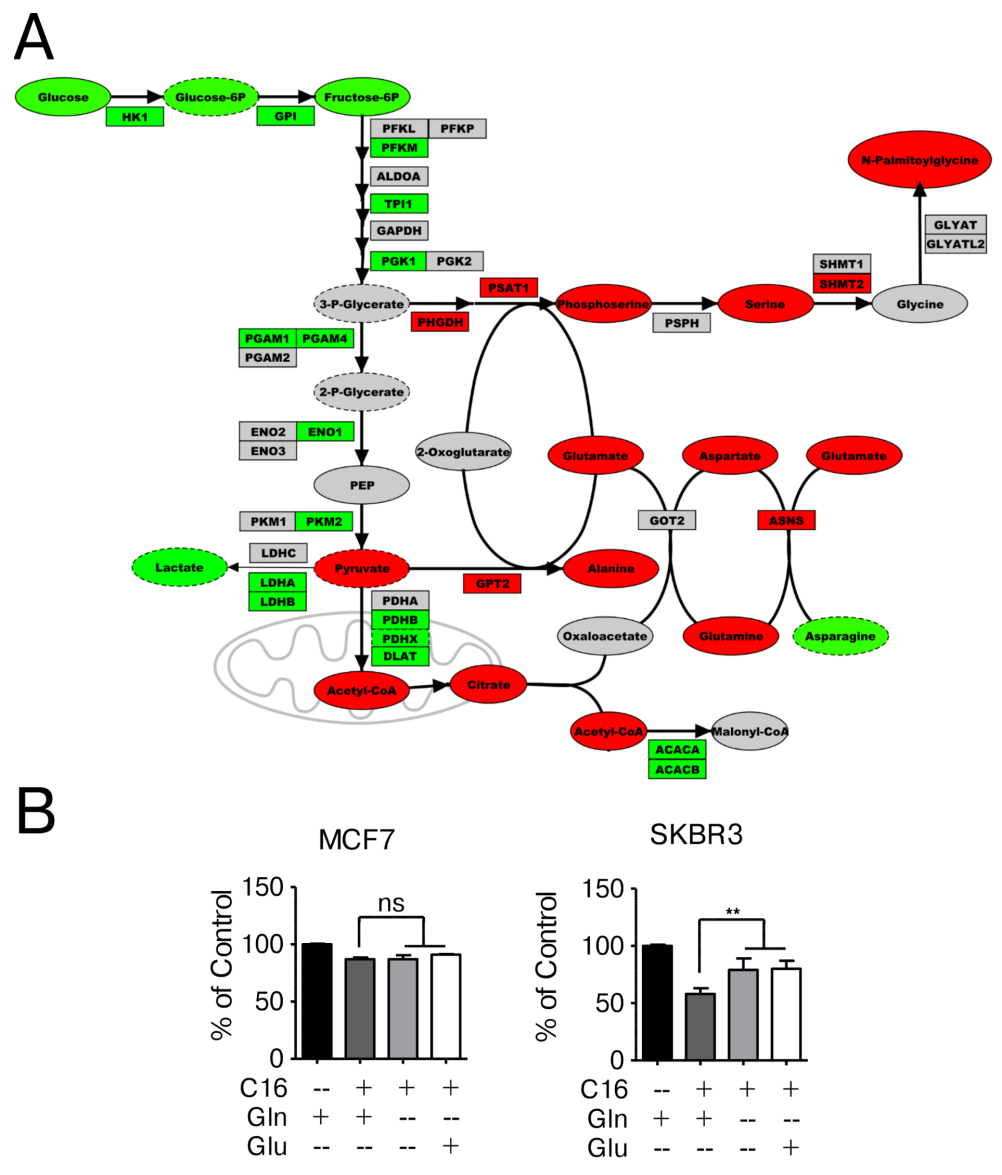
Network-overlay of metabolomic and transcriptomic data indicates alterations in glutamine, serine and glucose metabolism **(A)** Detailed section of a network-overlay of metabolomic and transcriptomic data of SKBR3 cells after 24 h treatment with 250 μM palmitate. Glycolysis and serine/glycine synthesis are connected to glutamine metabolism through a series of transamination reactions. Networks were created in Metscape, detailed section were ported into PathVisio and edited for clarity. Oval nodes indicate metabolites, boxes indicate genes. Red fill color denotes upregulation, green downregulation compared to control conditions. Grey nodes are part of the network but were not identified or did not change in abundance/expression. Dashed outline of a node indicates p ≤0.1, whereas solid outlines indicate p ≤ 0.05. **(B)** MCF7 and SKBR3 cells were treated with 250 μM palmitate for 24 h in full growth medium or drop-out medium without glutamine or medium containing glutamate instead of glutamine. After fixation, nuclei were stained with Hoechst 33342 and quantified using the INCell Analyzer 2200 and INCell Investigator software. The data were normalized to control conditions and are presented as mean ± SD. ^**^ = p ≤ 0.01, one-way ANOVA with Bonferroni post-test. n = 3.

Arginine, proline and glutamine/glutamate metabolism are tightly linked as all of these compounds share a central five carbon backbone. Glutamate is the required donor of amino groups in the biosynthesis of aspartate and alanine through transamination reactions. In addition to arginine and proline metabolism, glutamate, aspartate and alanine metabolism are identified as significantly enriched pathways after palmitate supplementation in HER2/neu-positive SKBR3 breast cancer cells (Figure 2B). Figure 5A shows a section of the gene-metabolite network encompassing glutamate, aspartate and alanine metabolism and associated metabolites. Palmitate supplementation results in increased levels of glutamine, glutamate, alanine and serine which are interconnected through transaminases that are also upregulated at the mRNA level. Of interest here is the distinct upregulation in SKBR3 cells of the serine/glycine synthesis pathway on the metabolite as well as the mRNA level of the enzymes PHGDH, PSAT1 and SHMT2.

**Figure 6:**
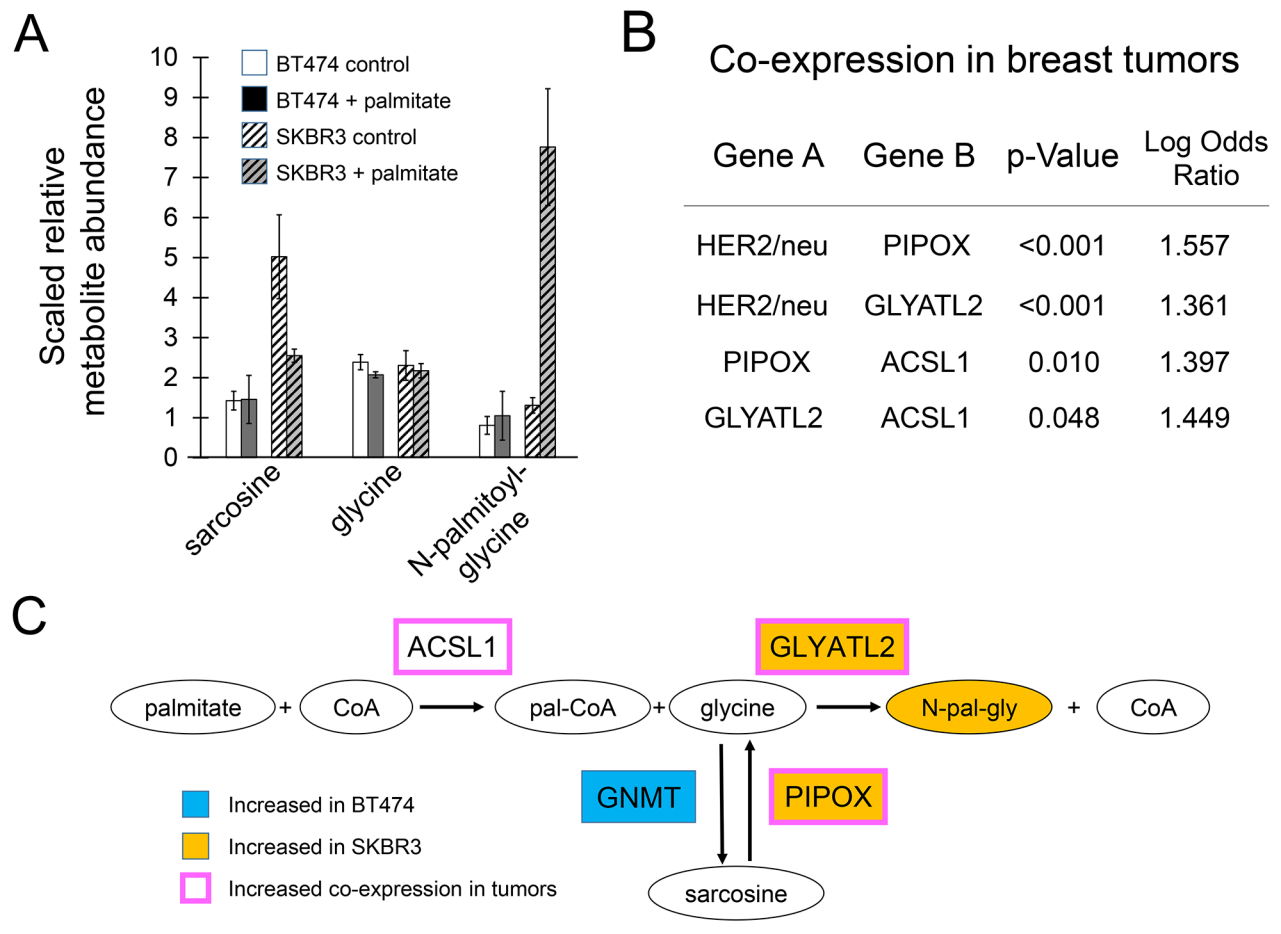
Metabolites related to N-palmitoylglycine production **(A)** Compound levels from metabolite profiling of palmitate-treated HER2/neu-positive BT474 and SKBR3 breast cancer cells. Cells were treated with 250μM palmitate or vehicle for 24 h in triplicate. Data are presented as mean ± SD. **(B)** Increased expression of PIPOX and GLYATL2 is associated with HER2/neu-positive breast cancer markers in molecular profiling data of 825 human breast tumors from the TCGA Breast Invasive Carcinoma project (http://cancergenome.nih.gov/). Analysis of mutual exclusivity and co-occurrence indicate statistically significant levels of co-occurrence of increased expression of genes encoding enzyme in the pathway producing N-palmitoylglycine in tumors with HER2/neu-positive signatures. **(C)** BT474 cells intrinsically overexpress GNMT. SKBR3 cells intrinsically overexpress GLYATL2 and have an amplification of PIPOX consistent with increased production of N-palmitoylglycine. When exposed to exogenous palmitate, SKBR3 cells display increased Oval nodes indicate metabolites, boxes indicate genes. Orange fill color denotes upregulation; blue downregulation.

The metabolite that changed the most in SKBR3 cells in both initial and secondary analyses was N-palmitoylglycine. In the initial analysis, this compound was upregulated 23-fold. Its reduced downstream metabolite, N-palmitoylethanolamide, was upregulated 12-fold. The pronounced increase in this compound suggests that sequestration of palmitate as a glycine adduct may represent a means of detoxification. Consistent with this notion, supplementing the growth media with N-palmitoylglycine at concentrations equal to that of toxicity-inducing palmitate shows no effects on growth or apoptotic rates of SKBR3 cells (Supplementary Figure 2). Although levels of these metabolites changed consistently and substantially in SKBR3 cells, in additional metabolomic experiments in BT474 and MCF7 cells the changes were less dramatic and not statistically significant. A possible explanation for this was noted in the HER2/neu positive breast cancer cell lines, SKBR3 and BT474 having differences in the pathway responsible for the production of glycine. Vehicle-treated SKBR3 cells have higher amounts of sarcosine compared to vehicle-treated BT474 cells (Figure 6A). Upon exposure to palmitate, the level of sarcosine is lowered two fold in SKBR3 cells while N-palmitoylglycine levels increase. Both sarcosine and N-palmitoylglycine levels remain essentially unchanged in BT474 cells. We theorize that the magnitude of the changes in SKBR3 cells is likely explained by underlying genetic differences although additional work is necessary to confirm this hypothesis. BT474 cells under normal conditions overexpress GNMT which catalyzes the conversion of glycine into sarcosine. SKBR3 cells overexpress GLYATL2 and have an amplification of PIPOX both of which are consistent with increased flux towards N-palmitoylglycine (Figure 6B). This may indicate that sequestration of palmitate as a glycine adduct occurs in the lipogenic SKBR3 cells under standard culture conditions. This may be a generalized feature of some HER2/neu positive breast cancers as analysis of expression of these genes in human breast cancers shows that PIPOX and GLYATL2 exhibit statistically significant tendencies to have increased expression co-occur with increased expression of HER2/neu/ERBB2 (p-value <0.001) in human breast tumors (Figure 6C). For each gene alone, 40-50% of the cases of upregulation in breast cancers occur in the HER2/neu positive subtype. Nevertheless, that this pathway is not upregulated in HER2/neu positive BT474 cells suggests that (i) it may not be a feature of all tumors and (ii) that some other mechanism for avoiding the toxicity of palmitate must occur in these cells.

In SKBR3 cells, downregulation of glycolysis is evident at the metabolite level in several steps and at the mRNA level of most enzymes throughout the pathway. High levels of acetyl-CoA and citrate, a potent allosteric inhibitor of glycolysis [33], are likely to be cytosolic as entry of pyruvate into the citrate cycle is diminished through the downregulation of the pyruvate dehydrogenase complex and upregulation of pyruvate dehydrogenase kinase 4 (PDK4) ([4], Supplementary Figure 3 and [14]. These changes are consistent with the inhibition of fatty acid synthesis and the accumulation of upstream metabolites. Previous studies have proposed a Warburg-like metabolic phenotype in HER2/neu-positive breast cancer that couples high glycolytic flux with fatty acid synthesis to regenerate reducing equivalents and shuttle carbons into fatty acids [6, 9, 10]. This metabolic phenotype necessitates a truncated TCA cycle based on constant export of citrate and creates a need for compensatory, anaplerotic reactions, e.g. glutaminolysis, to replenish TCA cycle intermediates [34]. The possibility that high glycolytic and glutaminolytic flux contribute to the growth-impairment of HER2/neu-positive SKBR3 cells in palmitate-supplemented media was tested by lowering the glucose and glutamine concentrations in the media and determining the effects on cell growth. Pre-adaptation of the cells to low glucose conditions reduces the glycolytic rate before the cells are exposed to palmitate. Indeed, we observe a significant increase in cell viability of SKBR3 cells in low glucose- or fructose-containing, palmitate-supplemented growth media (Supplementary Figure 4), suggesting that higher glycolytic activity exacerbates the effects of palmitate on the cells. This is likely due to a decreased need for NADH and NADPH oxidation required during glucose-based lipogenesis and the resulting decrease in endogenous palmitate production. Additionally, simplification of fuel utilization such that beta-oxidation may play a role under glucose-limited, normoxic conditions *in vitro* is possible. With these possible explanations, it is somewhat interesting that palmitate supplementationdoes not induce any significant changes in lactate levels.

Importantly, we observe a similar effect on increased survival when the cells are grown in palmitate-supplemented, glutamine-free media (Figure 5). The omission of glutamine in the growth media significantly ameliorates the negative effects of palmitate in SKBR3 cells, however the protective effect is not absolute. This is expected since the effects of palmitate are likely to be multifactorial. Interestingly, substituting glutamate for glutamine in the growth media yields identical results in SKBR3 cells as in glutamine-free media, indicating that the carbon backbone of glutamine may not be the contributing factor but rather the γ-amino group on glutamine. This is particularly interesting as arginine deprivation also alleviates the negative effects of palmitate supplementation (Figure 4), since both conditions effectively limit the amount of nitrogen in the system. No changes are observed in the response of MCF7 cells to palmitate under low glucose/fructose or glutamine-free growth conditions (Figure 5B and Supplementary Figure 4).

### Limiting glutamine availability attenuates palmitate-induced ER-stress

**Figure 7:**
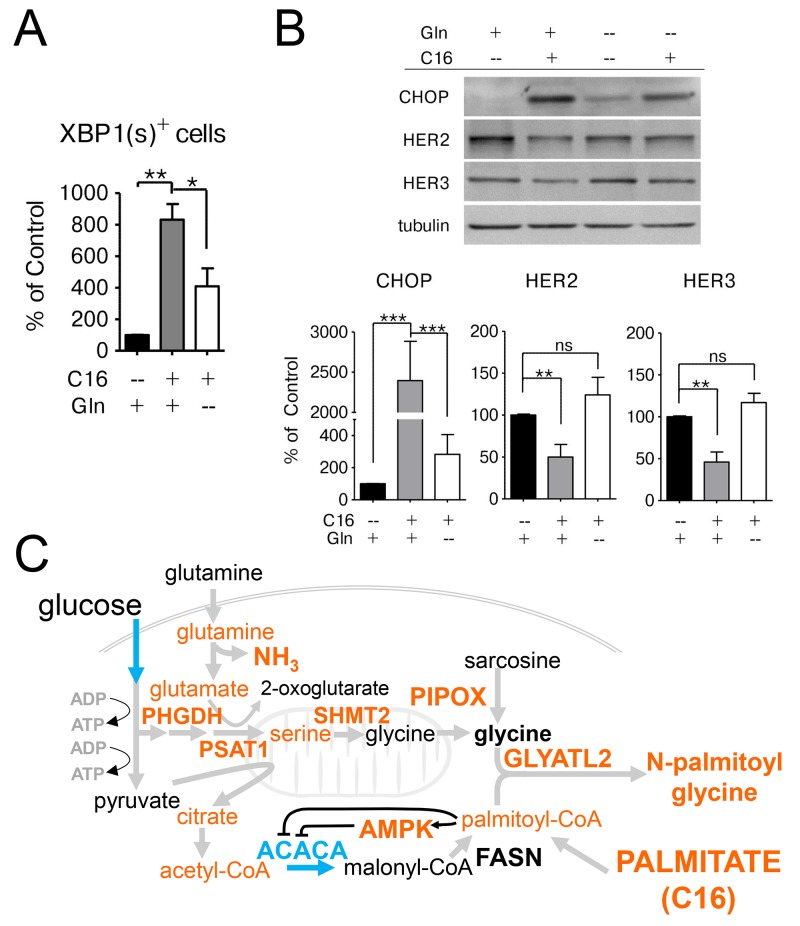
Glutamine supplementation exacerbates the lipotoxic effects of palmitate in HER2/neu-positive SKBR3 cells **(A)** SKBR3 cells were transfected with the transcriptional reporter construct pCAX-XBP1-ΔDBD-venus which results in the expression of a functional fluorescent XBP1-venus fusion protein after mRNA splicing by the ER-stress regulator IRE1 as described in [14, 35]. The cells were treated with 250 μM palmitate 24 h post-transfection, either in full growth medium, drop-out medium without glutamine or drop-out medium with glutamate instead of glutamine. Green fluorescent cells, that are indicative of XBP1-splicing and ER-stress activation, were counted using the InCell Analyzer 2200. **(B)** SKBR3 cells were treated with 250 μM palmitate or vehicle for 24 h either in full growth medium or drop-out medium without glutamine. Total protein was harvested and subjected to western blot analysis to evaluate the protein expression changes for the ER-stress related marker DDIT3/CHOP and the growth factor receptors HER2 and HER3. Each experiment was performed in triplicate, representative images are shown. Signal was quantified using ImageJ. Intensities were normalized to loading control and vehicle-control. The data is presented as mean ± SD. ^*^ = p ≤ 0.05, ^**^ = p ≤ 0.01, ^***^ = p ≤ 0.001, one-way ANOVA with Bonferroni post-test (A), Student’s *t*-test (B). n = 3. **(C)** Overview of palmitate induced lipotoxicity in SKBR3 cells. Exogenous palmitate application results in increased activity in glycine production and decreased fatty acid synthesis. Glycine production is accomplished by transamination reactions. Fatty acid synthesis down regulation occurs through activation of AMPK. Metabolites and enzymes increased abundance are depicted in orange. Decreases in ACACA activity and early glycolytic pathway metabolites are depicted in blue.

We have recently reported that palmitate supplementation induces an ER-stress response in HER2/neu-positive SKBR3 cells, which is accompanied by IRE1-mediated XBP1-splicing and upregulation of the pro-apoptotic regulator CHOP and results in CHOP-dependent apoptosis [14]. We investigated whether the metabolic changes we observed after palmitate supplementation play a role in the activation of the ER-stress response. We used a transcriptional reporter construct described by Samali et al. to evaluate whether glutamine availability contributes to palmitate-induce XBP1 splicing [35]. SKBR3 cells were transfected with the pCAX-XBP1-ΔDMD-venus vector which results in the expression of a functional fluorescent XBP1-venus fusion protein if the XBP1 mRNA is spliced by the ER-stress activator IRE1. The DNA-binding domain has been deleted from the reporter construct to prevent the fusion-protein from interfering with the function of the endogenous XBP1(s) protein. Twenty-four hours post-transfection the cells were switched to palmitate-supplemented growth media containing either the normal glutamine concentration or no glutamine. As expected, palmitate supplementation causes an increase in XBP1(s)-positive cells. This response is significantly attenuated if glutamine availability is limited, however there is still a significant difference compared to control conditions, indicating that other factors besides glutamine contribute to the induction of ER-stress (Figure 7A). This is corroborated by our results showing that glutamine starvation also significantly reduces the palmitate-mediated induction of CHOP (Figure 7B).

Palmitate supplementation also leads to a disruption of growth factor signaling through the reduced expression of HER2 and HER3 at the protein level [14]. Culturing HER2/neu-positive SKBR3 breast cancer cells in glutamine-free, palmitate-supplemented growth media restores the expression of HER2 and HER3 (Figure 7B). These data indicate that the negative effects of palmitate supplementation in SKBR3 cells are in part mediated through glutamine metabolism.

## DISCUSSION

HER2/neu-positive breast cancer cells have been shown to exhibit a pro-lipogenic phenotype. These cells display high levels of endogenous saturated fatty acids and neutral lipids and exhibit a Warburg-like physiology that relies on active fatty acid synthesis for survival and aggressive behavior [6, 8-10, 21]. However this phenotype may represent an Achilles heel as exogenously supplied palmitate is toxic to HER2/neu-positive breast cancer cells [8, 14]. In this study, we used physiological concentrations of exogenous palmitate to model high levels of dietary saturated fat intake and investigated the metabolic changes associated with the lipotoxicity observed in HER2/neu-positive breast cancer cells and how they relate to their Warburg-like physiology. Our global metabolite profiling analysis identified glutamine metabolism as a novel mediator of palmitate-induced lipotoxicity in the HER2/neu-positive SKBR3 breast cancer cell line (Figure 2 and 5).

Given the altered metabolic phenotype of HER2/neu-positive breast cancer cells, our data suggest that exogenous palmitate exerts its toxic effects by interfering with FA synthesis (Figure 7C). Upon uptake into the cell, palmitate will be converted into palmitoyl-CoA before it can be further metabolized. Co-administration of palmitate and oleate shows no toxic effect in either cell line, even though the total FA concentration is higher in this case. This effect has been previously observed in CHO cells [36], rat beta-cells [37], rat islets [38, 39] and human beta cells [40]. It has been suggested that combining palmitate and oleate facilitates the sequestration of these FAs into TAGs and thus reduces their impact on pathways that induce apoptosis [13]. This suggests that palmitoyl-CoA may mediate some of the toxic effects in SKBR3 cells (Figure 7C). Palmitoyl-CoA has been shown to be a major allosteric inhibitor of ACACA/ACC, which catalyzes the rate-limiting step of fatty acid synthesis, in a classic example of product feedback inhibition [41]. This inhibition is exacerbated by the activation of AMPK which inactivates ACC through phosphorylation (Figure 3) and results in the accumulation of the upstream metabolites citrate and acetyl-CoA (Figure 5 and Supplementary Table 2). Citrate is an allosteric inhibitor of glycolysis and an allosteric activator of ACACA/ACC, however the binding affinity of ACC for citrate is greatly reduced by AMPK-dependent phosphorylation [33, 42]. The fact that inhibition of FA synthesis by palmitate feeds back into glycolysis as the major energy producing pathway in these cells is evident through the concerted downregulation of almost all glycolytic enzymes and metabolic intermediates as wells as a significant increase in adenosine monophosphate (AMP, HMDB00045) (Figure 5 and Supplementary Table 1). Reducing the glycolytic activity in the cells by limiting glucose availability significantly ameliorates the toxic effects of palmitate supplementation in HER2/neu-positive SKBR3 breast cancer cells (Supplementary Figure 4). This is likely due to reducing demands on anaplerotic reactions and the amount of palmitate produced although additional work will be needed to confirm these explanations.

As previously stated, this Warburg-like physiology that shuttles glucose carbons through a truncated TCA cycle into lipid precursors [6, 9, 10], requires compensatory, anaplerotic reactions to replenish TCA cycle intermediates. Glutamine metabolism has been shown to provide a carbon source that facilitates the cells’ ability to use glucose-derived carbons and TCA cycle intermediates as biosynthetic precursors [34]. Among all breast cancer subtypes, HER2-positive tumors show the highest expression of glutaminase (GLS) and glutamate dehydrogenase (GDH) [43] and overexpression of HER2 in a cell line model directly increases GLS expression [44]. Anaplerotic glutamine metabolism generates free ammonia through the hydrolysis of the γ-amino group on glutamine and is mostly secreted from the cell by an unknown mechanism [45]. Without rapid clearance of ammonia, intracellular concentrations may quickly increase to toxic levels [46]. Considering that the γ-amino group of glutamine seems to be the contributing factor to palmitate-induced cytotoxicity (Figure 4B) and growth in palmitate-supplemented media significantly increases the acetylated polyamine content (Figure 4A), it is conceivable that the generation of free ammonia contributes to the toxicity phenotype. Sequestration of palmitate as N-palmitoylglycine and the upregulation of the serine/glycine synthesis pathway (Figure 6) may be responsible for an increased demand on glutamate, which can only be produced through glutaminolysis. The transamination reactions required for serine/glycine production utilize the α-amino group of glutamate, rather than the γ-amino group of glutamine [47]. Similar to our observations for palmitate, ammonia treatment has been shown to activate AMPK, initiate ER-stress/UPR signaling and induce the expression of the pro-apoptotic regulator CHOP [14, 48, 49] (Figure 7). Culturing the cells in palmitate-supplemented, glutamine-free media significantly ameliorates the induction of ER-stress, as evidenced by reduced XBP1-splicing and CHOP expression (Figure 7A, 7B). Interestingly, the chemical chaperone 4-phenyl butyric acid (4-PBA) which, as we have previously reported, ameliorates palmitate-induced cytotoxicity in HER2/neu positive breast cancer cells, is also used in patients with urea cycle disorders to sequester excess ammonia [50].

These effects could conceivably extend to growth signaling since several studies have shown that HER2, fatty acid synthesis, ER-stress and glycolytic activity are functionally linked. ACC and FASN are upregulated in HER2/neu-positive breast cancers at the transcriptional [6] and the translational level [7] in a HER2-amplicon or HER2-dependent manner, respectively. Overexpression of FASN in immortalized non-tumorigenic mammary epithelial cells has been shown to induce HER2 overexpression and activation, which could be reversed by inhibitors of FASN [51]. Pharmacological inhibition of FASN induces ER-stress [52], decreases HER2 protein levels [53, 54], sensitizes cells to trastuzumab treatment [54] and reverses acquired autoresistance to trastuzumab [51, 55]. Similarly, HER2 expression has been shown to be regulated by glucose availability in an ER-stress-dependent manner [56]. These data are in line with our observations that exogenous palmitate inhibits FA synthesis, reduces glycolytic activity, induces ER-stress, decreases HER2 and HER3 protein levels and sensitizes the cells to trastuzumab-mediated growth inhibition [14]. It is interesting to note that this palmitate-mediated reduction in HER2 and HER3 expression is significantly inhibited under glutamine-free growth conditions (Figure 6), suggesting that metabolic changes in the cells may be contributing to and constraining alterations in oncogenic growth signaling, a concept reviewed in [57]. This study provides further evidence, that the Warburg-like physiology of HER2/neu-positive breast cancer cells creates a distinct metabolic program that is reliant upon, and feeds back into, the HER2 signaling network.

The production of N-palmitoylglycine as the major palmitate-derived metabolite in HER2/neu-positive breast cancer cells is especially curious and warrants further investigation. N-acyl amides are a relatively novel group of endogenous bioactive lipids that have not been very well characterized and may act through a variety of G-protein coupled receptors as well as cannabinoid receptors to influence various endogenous signaling processes [58–60]. Generation of these bioactive lipids by cancer cells has not been previously reported and may have implications for the tumor microenvironment as well as for systemic responses to the tumor.

We have previously shown that physiological concentrations of exogenous palmitate as a model for dietary saturated fat intake induce a partial ER-stress response and CHOP-dependent apoptosis in HER2/neu-positive breast cancer cells, which is accompanied by a significant reduction in HER2 and HER3 protein levels and sensitizes cells to trastuzumab treatment [14]. Here we investigated the metabolic implications of this response and reveal glutamine metabolism as a novel important mediator of saturated fat-induced lipotoxicity in HER2/neu-positive breast cancer cells. In our cell line model, metabolic changes are not only induced by oncogenic alterations but are creating constraints that feed back into oncogenic signaling. Based on our results, it is intriguing to speculate that high levels of dietary saturated fatty acids are capable of interfering with HER2/HER3 expression and the associated metabolic phenotype during disease development. The lipotoxicity effect investigated here is most likely due to a combination of genetic alterations that exist in HER2/neu-positive breast cancer cells, considering that the HER2 amplicon has been shown to comprise several genes in a large section on chromosome 17 [3, 4, 15–19] and other genes have been shown to be co-overexpressed and required for breast cancer cell survival [6]. Initial results in our lab indicate that overexpression of HER2 alone is not sufficient to induce sensitivity to palmitate. Obesity is a well described risk factor for a number of pathologies, one of which is breast cancer. Studies have commonly looked at the correlation between body mass index (BMI) and a number of statistics, including cancer incidence and survival. However, no significant correlation has been reported linking high BMI and HER2/neu expression [10]. Recently, Sieri et al. reported the results of a new study that followed 337,327 women for 11.5 years and evaluated dietary fat intake as a predictor of developing breast cancer. A diet high in saturated fatty acids was found to be positively associated with the development of HER2/neu-negative disease, but not HER2/neu-positive disease [20]. Given the rather modest effects shown in these epidemiological studies and the common sequelae of a diet high in saturated fat, however, palmitate consumption is not envisioned as a therapy. Nevertheless, further investigation of this phenomenon may lead to an improved understanding of breast cancer cell physiology and identify new therapeutic targets. Finally, the production of the bioactive lipid N-palmitoylglycine has not previously been reported in any cancer type and is particularly interesting with regards to tumor-induced paracrine and endocrine signaling. Further research is needed to elucidate the role of saturated fatty acids and their associated N-acyl glycine derivatives in the etiology of breast cancer.

## MATERIALS AND METHODS

### Cell culture and chemicals

Breast cancer cell lines SKBR3, BT474, HCC1569, MCF7 and MCF10A were obtained from the American Type Culture Collection (Manassas, VA, USA). Cells were cultured in Dulbecco’s modified Eagle’s medium (DMEM, Hyclone, Logan, UT) supplemented with 10% fetal bovine serum (Hyclone) at 37°C and 5% CO2. MCF10A cells were cultured and infected with pLXSN-neu or vector control retroviruses as previously described [6]. All cell lines were authenticated in March 2016 by the SUNY-Albany Center for Functional Genomics Molecular Core Facility using a short tandem repeat method (Promega GenePrint 10 system). Sodium palmitate, etomoxir and fructose were obtained from Sigma-Aldrich (St. Louis, MO), palmitoyglycine was obtained from Cayman Chemical (Ann Arbor, MI). All individual components needed to prepare custom DMEM culture media according to the composition list provided by Hyclone, were obtained from Sigma-Aldrich or Thermo Fisher Scientific Inc. (Waltham, MA). Drop-out media were prepared as needed, sterile filtered before usage and supplemented with 10% dialyzed FBS (Hyclone). For drop-out experiments, cells were acclimated to the new media for 24 h prior to treatment. Palmitate was solubilized in ethanol and diluted in full growth medium (DMEM, 10% FBS) prior to treatment of cells. The palmitate concentration was chosen based on the sensitivity profiles of the different HER2/neu-positive breast cancer cell lines. 250 μM palmitate leads to a 70-80 % reduction in viability in SKBR3 cells. According to the literature, fasting FFA concentrations in plasma/serum are in the range of 300-600μM [61, 62] [63] with palmitate representing about one quarter of the total FFAs [64, 65]. However, these concentrations vary extensively based on the analysis method used for quantification [65]. Based on these studies, 250 μM palmitate was deemed to be in the physiological range. All solutions were prepared immediately before usage. For nuclei counts, cells were plated in 96-well plates and allowed to adhere overnight. After treatment and incubation cells were fixed with 2.5 % formaldehyde and nuclei were stained with 1μg/mL Hoechst 33342 (Life Technologies, Grand Island, NY). Images of cells were acquired using an INCell Analyzer 2200 high-content imaging system and images were analyzed using the INCell Investigator software (GE Healthcare, Piscataway, NJ).

### Metabolite profiling

Global metabolite profiling was performed by Metabolon, Inc. (Durham, NC, USA), a commercial supplier of metabolic analyses using a proprietary platform that integrates the chemical analysis, identification and relative quantification as well as data-reduction and quality-control [66]. Samples were collected by trypsinization after 24 h of treatment with 250 μM palmitate or vehicle control. Cells pellets were stored at -80°C until all biological replicates were collected. The sample preparation process was carried out using the automated MicroLab STAR system from Hamilton Company. Recovery standards were added prior to the first step in the extraction process for QC purposes. Sample preparation was conducted using a proprietary series of organic and aqueous extractions to remove the protein fraction while allowing maximum recovery of small molecules. The resulting extract was divided into two fractions; one for analysis by LC and one for analysis by GC. Samples were placed briefly on a TurboVap (Zymark) to remove the organic solvent. Each sample was then frozen and dried under vacuum. Samples were then prepared for the appropriate instrument, either LC/MS or GC/MS. Following normalization to Bradford protein concentration, median scaling, imputation of missing values, if any, with the minimum observed value for each compound and log transformation of median scaled data, Welch’s two-sample *t*-test was used to identify biochemicals that differed significantly between experimental groups. The initial analysis was carried out with five biological replicates of SKBR3 cells. A second analysis was carried using three biological replicates each of SKBR3, BT474, MCF7 cells and MCF10A cells transduced with LXSN virus either empty or containing wild-type or activated rat neu [14]. The magnitude of changes in the second analysis was less than the first analysis though effects were largely similar (see Supplementary Materials).

### Metabolic assays

For detection of neutral fat stores, cells were stained with 1 μg/ml 4,4-difluoro-1,3,5,7,8-pentamethyl-4-bora-3a,4a-diaza-s-indacence (BODIPY 493/503) or LipidTOX Deep Red (Life Technologies, Grand Island, NY). Cells were grown in 96-well plates, fixed with 2.5% formaldehyde, stained with 1μg/ml BODIPY 493/503 or LipidTOX and counterstained for nuclei with 1μg/mL Hoechst 33342. Cells were imaged using the INCell Analyzer 2200 (GE Healthcare, Piscataway, NJ) high-content imaging system. Images were analyzed using the INCell Investigator software.

### Immunoblotting

Immunoblots were performed using standard protocols. Cells were lysed directly in Laemmli loading buffer, proteins were resolved by SDS-PAGE and transferred to PVDF membranes (EMD Millipore, Billerica, MA). Membranes were blocked in Tris buffered saline (TBS) containing 0.1% Tween and 5% non-fat powdered milk (TBS-T, 5% milk). Primary antibody incubation was performed in TBS-T, 5% milk overnight at 4°C. Proteins were visualized using a species-specific HRP-conjugated secondary antibody and the ECL Plus chemifluorescent detection system (Thermo Fisher Scientific Inc., Waltham, MA) on a STORM scanner ((GE Healthcare, Piscataway, NJ). Signal was quantified using ImageJ software (http://rsb.info.nih.gov/ij/). The following primary antibodies were used: ACC (#3676, Cell Signaling), phospho-ACC (Ser79, #3661, Cell Signaling), AMPKα (#2603, Cell Signaling), phospho-AMPKα (Thr172, #2535, Cell Signaling), DDIT3/CHOP (#2895, Cell Signaling), HER2 (#4290, Cell Signaling), HER3 (#4754, Cell Signaling), α-tubulin (MCA78G, AbD Serotec, Oxford, UK). Secondary antibodies: goat-anti-rabbit-HRP (#7074, Cell Signaling), horse-anti-mouse-HRP (#7076, Cell Signaling), goat-anti-rat-HRP (sc-2303), Santa Cruz Biotechnology, Inc., Santa Cruz, CA).

### Computational analyses

Metabolite pathway enrichment was performed using the web resource at www.metaboanalyst.ca [23, 24]. Metabolites were mapped to database identifiers and subsequently subjected to pathway enrichment analysis using the GlobalTest algorithm. Node importance for pathway topology analysis was determined using the “relative betweenness centrality" metric. Cytoscape (www.cytoscape.org) was used to visualize networks [29]. The Cytoscape plugin Metscape (http://metscape.ncibi.org) [30, 31] was used to overlay the global metabolite profiling results described in this study onto the microarray data (ArrayExpress: E-MTAB-2601) described in [14]. Pathways of interest were ported into PathVisio (http://www.pathvisio.org) to improve structure and visibility [67]. Heatmaps were generated using Cluster 3.0 [68] and visualized using TreeView [69].

### Transfections and reporters

For pCAX-XBP1-ΔDBD-venus reporter construct assays [35], cells were seeded in 96-well plates and allowed to adhere overnight before they were transfected using XtremeGene HP (Roche), according to the manufacturer’s instructions. Cells were treated as indicated in the individual experiments, 24 h post-transfection. Expression of the fluorescent protein is indicative of IRE1-mediated XBP1 splicing. The pCAX-XBP1-ΔDBD-venus reporter construct was a generous gift from Dr. Masayuki Miura, University of Tokyo. Green fluorescent cells were counted using the INCell Analyzer 2200 and INCell Investigator software (GE HEalthcare, Piscataway, NJ).

## SUPPLEMENTARY MATERIALS FIGURES AND TABLES





## Abbreviations

4-PBA: 4-phenylbutyric acid
ACC: acetyl-CoA carboxylase alpha
ACLY: ATP-citrate lyase
Ala: alanine
AMPK: AMP-activated protein kinase
Arg: arginine
Asp: aspartate
C16: palmitate
C18: stearate
C18:1: oleate
Ctrl: control
DPBS: Dulbecco’s phosphate buffered saline
FA: fatty acid
FASN: fatty acid synthase
FBS: fetal bovine serum
Gln: glutamine
Glu: glutamate
GSEA: gene set enrichment analysis
HMDB: human metabolome database
HMECs: human mammary epithelial cells
HRP: horseradish peroxidase
PBP: PPARγ binding protein
PERK: PKR-like endoplasmic reticulum kinase
SAM: S-adenosyl methionine
SDS-PAGE: sodium dodecylsulfate polyacrylamide gel electrophoresis
TAG: triacylglycerides
TBS: Tris buffered saline
UPR: unfolded protein response
