# Supplementary material for: Global metabolite profiling analysis of lipotoxicity in HER2/neu-positive breast cancer cells
Source: Oncotarget. 2018 Jun 5;9(43):27133–50. doi: 10.18632/oncotarget.25500 (PMC6007458; doi:10.18632/oncotarget.25500)
Supplement: Supplementary file 1 [file oncotarget-09-27133-s001.pdf]

Global metabolite profiling analysis of lipotoxicity in HER2/neu-positive breast cancer cells

SUPPLEMENTARY MATERIALS

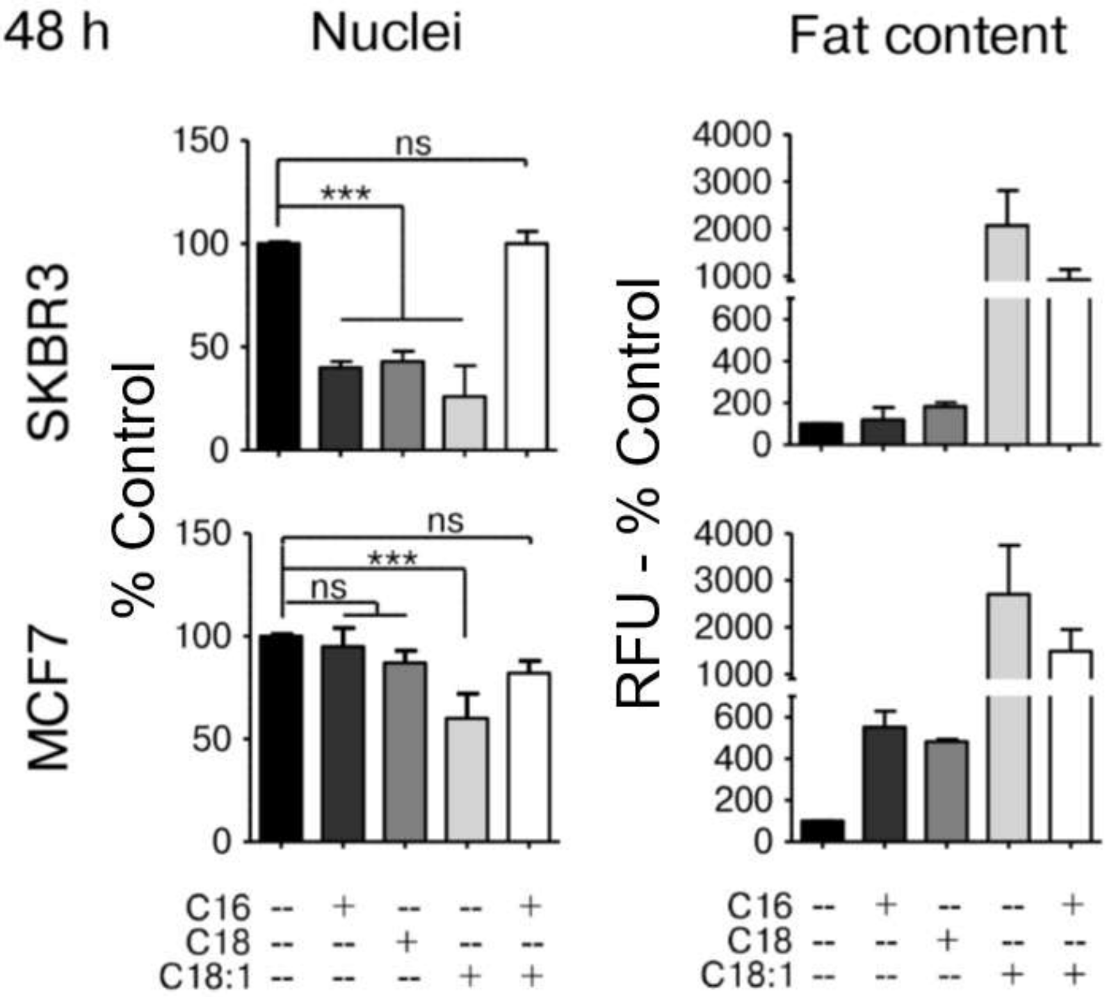

Supplementary Figure 1: HER2/neu-positive SKBR3 and HER2-normal MCF7 cells differ in their response to saturated fatty acids. HER2/neu-positive SKBR3 and HER2-normal MCF7 breast cancer cells were treated with 250 µM palmitate, stearate, oleate, palmitate + oleate or vehicle for 48 h. Cells were fixed and neutral lipids were stained with BODIPY 493/503. Nuclei were stained with Hoechst 33342. BODIPY fluorescence and nuclei were imaged and quantified using the INCell Analyzer 2200 and INCell Investigator software. Fluorescence intensity per cell is proportional to the neutral lipid content in the cell. Statistical analysis was carried out in Graphpad Prism. Data are presented mean ± SD. $^{*} = p \leq 0.05$, one-way ANOVA with Bonferroni post-test, n~=~3.

Supplementary Table 1: Metabolite concentration table used as input for the bioinformatics resource at metaboanalyst.ca. Metabolites with significantly altered concentrations in SKBR3 cells after 24hrs of treatment with cells after 24h treatment with 250 µM palmitate (C16_24 samples) or vehicle control (VEH_24 samples). Metabolites are listed by Human Metabolome Database identification number.

See Supplementary File 1

Supplementary Table 2: Detailed results of the pathway enrichment analysis for SKBR3 cells treated with palmitate

	Total Cmpd	Hits	Raw p	-log(p)	Holm adjust	FDR	Impact
Fatty acid metabolism	50	4	4.28E-09	1.93E+01	2.44E-07	2.44E-07	0.24
Arginine and proline metabolism	77	10	3.95E-07	1.47E+01	2.21E-05	1.13E-05	0.39
Ascorbate and aldarate metabolism	45	4	1.28E-06	1.36E+01	7.03E-05	2.43E-05	0.10
Pyrimidine metabolism	60	7	2.18E-06	1.30E+01	1.18E-04	3.11E-05	0.19
Nitrogen metabolism	39	10	9.30E-06	1.16E+01	4.93E-04	1.02E-04	0.01
Amino sugar and nucleotide sugar metabolism	88	6	1.13E-05	1.14E+01	5.86E-04	1.02E-04	0.18
Phenylalanine, tyrosine and tryptophan biosynthesis	27	3	1.29E-05	1.13E+01	6.56E-04	1.02E-04	0.01
Pentose and glucuronate interconversions	53	8	1.54E-05	1.11E+01	7.70E-04	1.02E-04	0.08
Glutathione metabolism	38	10	1.61E-05	1.10E+01	7.87E-04	1.02E-04	0.39
Starch and sucrose metabolism	50	5	1.95E-05	1.08E+01	9.38E-04	1.06E-04	0.26
Tyrosine metabolism	76	2	2.21E-05	1.07E+01	1.04E-03	1.06E-04	0.05
Pantothenate and CoA biosynthesis	27	8	2.23E-05	1.07E+01	1.04E-03	1.06E-04	0.42
Tryptophan metabolism	79	3	3.00E-05	1.04E+01	1.35E-03	1.31E-04	0.11
Glycerophospholipid metabolism	39	3	4.18E-05	1.01E+01	1.84E-03	1.70E-04	0.19
Purine metabolism	92	8	5.29E-05	9.85E+00	2.28E-03	2.01E-04	0.19
Valine, leucine and isoleucine biosynthesis	27	7	6.03E-05	9.72E+00	2.53E-03	2.15E-04	0.15
beta-Alanine metabolism	28	6	6.71E-05	9.61E+00	2.75E-03	2.25E-04	0.33
Alanine, aspartate and glutamate metabolism	24	4	8.13E-05	9.42E+00	3.25E-03	2.57E-04	0.69
Thiamine metabolism	24	1	9.13E-05	9.30E+00	3.56E-03	2.74E-04	0.00
One carbon pool by folate	9	1	1.14E-04	9.08E+00	4.35E-03	3.26E-04	0.00
Sphingolipid metabolism	25	2	1.33E-04	8.92E+00	4.93E-03	3.61E-04	0.01
Galactose metabolism	41	5	1.44E-04	8.85E+00	5.19E-03	3.73E-04	0.06
Cysteine and methionine metabolism	56	7	1.51E-04	8.80E+00	5.29E-03	3.74E-04	0.11
Inositol phosphate metabolism	39	4	1.74E-04	8.66E+00	5.90E-03	4.12E-04	0.18
Phenylalanine metabolism	45	2	2.62E-04	8.25E+00	8.66E-03	5.91E-04	0.12
Fatty acid biosynthesis	49	6	2.70E-04	8.22E+00	8.66E-03	5.91E-04	0.02
Methane metabolism	34	4	3.01E-04	8.11E+00	9.34E-03	6.20E-04	0.10
Butanoate metabolism	40	2	3.05E-04	8.10E+00	9.34E-03	6.20E-04	0.07
Glycolysis or Gluconeogenesis	31	3	3.29E-04	8.02E+00	9.55E-03	6.29E-04	0.04
Aminoacyl-tRNA biosynthesis	75	17	3.31E-04	8.01E+00	9.55E-03	6.29E-04	0.23
Glycine, serine and threonine metabolism	48	7	3.42E-04	7.98E+00	9.55E-03	6.30E-04	0.31
Cyanoamino acid metabolism	16	3	5.41E-04	7.52E+00	1.41E-02	9.64E-04	0.00
D-Glutamine and D-glutamate metabolism	11	2	5.77E-04	7.46E+00	1.44E-02	9.97E-04	0.14
Valine, leucine and isoleucine degradation	40	7	8.53E-04	7.07E+00	2.05E-02	1.43E-03	0.13
Histidine metabolism	44	4	1.69E-03	6.38E+00	3.88E-02	2.75E-03	0.14
Fatty acid elongation in mitochondria	27	2	1.87E-03	6.28E+00	4.12E-02	2.96E-03	0.27
Lysine biosynthesis	32	3	3.02E-03	5.80E+00	6.34E-02	4.65E-03	0.10
D-Arginine and D-ornithine metabolism	8	1	3.51E-03	5.65E+00	7.03E-02	5.27E-03	0.00
Porphyrin and chlorophyll metabolism	104	2	3.69E-03	5.60E+00	7.03E-02	5.39E-03	0.00
Pentose phosphate pathway	32	2	4.39E-03	5.43E+00	7.90E-02	6.25E-03	0.09
Steroid hormone biosynthesis	99	1	4.89E-03	5.32E+00	8.31E-02	6.53E-03	0.00
Citrate cycle (TCA cycle)	20	3	4.89E-03	5.32E+00	8.31E-02	6.53E-03	0.15
Taurine and hypotaurine metabolism	20	3	4.92E-03	5.31E+00	8.31E-02	6.53E-03	0.41
Propanoate metabolism	35	4	5.66E-03	5.17E+00	8.31E-02	7.20E-03	0.14
Ubiquinone and other terpenoid-quinone biosynthesis	36	3	5.68E-03	5.17E+00	8.31E-02	7.20E-03	0.04
Nicotinate and nicotinamide metabolism	44	3	5.86E-03	5.14E+00	8.31E-02	7.26E-03	0.05
Glycerolipid metabolism	32	1	6.05E-03	5.11E+00	8.31E-02	7.34E-03	0.19
Primary bile acid biosynthesis	47	2	6.18E-03	5.09E+00	8.31E-02	7.34E-03	0.06
Lysine degradation	47	2	7.85E-03	4.85E+00	8.31E-02	9.13E-03	0.15
Pyruvate metabolism	32	1	9.54E-03	4.65E+00	8.31E-02	1.05E-02	0.25
Synthesis and degradation of ketone bodies	6	1	9.54E-03	4.65E+00	8.31E-02	1.05E-02	0.00
Terpenoid backbone biosynthesis	33	1	9.54E-03	4.65E+00	8.31E-02	1.05E-02	0.00
Sulfur metabolism	18	1	1.08E-02	4.53E+00	8.31E-02	1.16E-02	0.00
Fructose and mannose metabolism	48	1	1.74E-02	4.05E+00	8.31E-02	1.84E-02	0.03
Riboflavin metabolism	21	2	3.01E-02	3.50E+00	9.04E-02	3.12E-02	0.00
Glyoxylate and dicarboxylate metabolism	50	3	3.89E-02	3.25E+00	9.04E-02	3.96E-02	0.01
Biotin metabolism	11	1	4.34E-02	3.14E+00	9.04E-02	4.34E-02	0.00

Metabolite pathway enrichment was performed using the web resource at www.metaboanalyst.ca. Metabolites were mapped to database identifiers and subsequently subjected to pathway enrichment analysis using the GlobalTest algorithm. Pathway topology analysis was performed using the "relative betweenness centrality" metric.

Supplementary Table 3: Comparison of relevant metabolite abundance changes in initial SKBR# study and second study involving additional cell lines

	SKBR3 study 1	SKBR3 study 2	BT474 study 2	MCF7 study 2
glutamine	1.55	1.72	1.1	0.96
glutamate	1.21	1.46	1.12	1.24
aspartate	1.25	1.68	1.1	1.5
putrescine	1.38	0.9	0.93	1.11
spermidine	1.41	1.84	1.41	1.36
methylthioadenosine	1.81	1.78	1.24	1.3
arginine	1.19	1.54	1.21	1.24
proline	1.29	1.46	1.18	1.24
alanine	1.09	1.41	1.03	1.23
glycine	1.03	1.26	1.14	1.12
serine	1.19	1.38	1.04	1.17
N-palmitoylglycine	22.18	7.84	1.29	2.07
palmitoylethanolamide	12.82	2.85	1.19	2.03

Fold change of scaled relative metabolite abundance after palmitate treatment. Red shading indicates $p \leq 0.05$; pink shading $p \leq 0.1$.

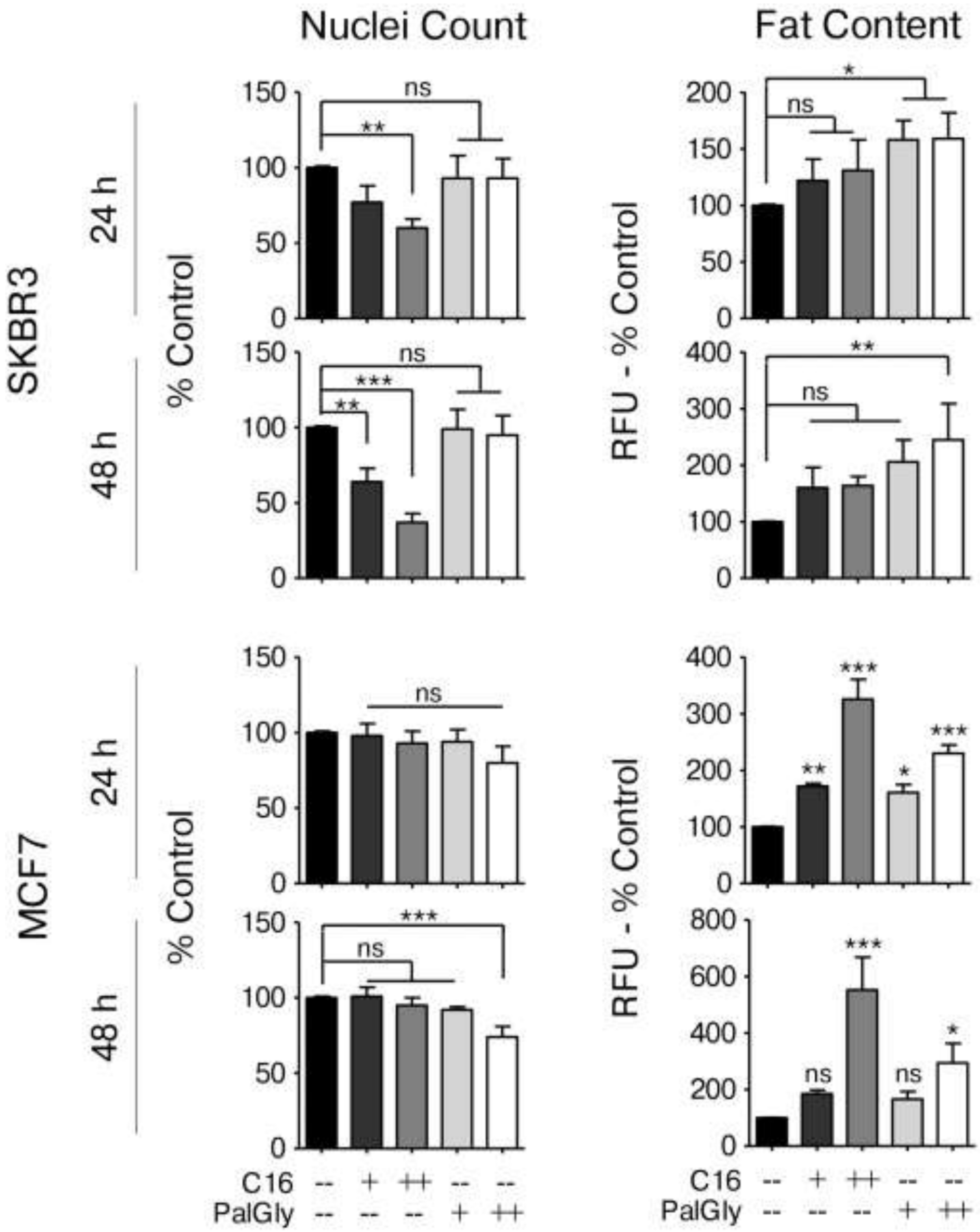

Supplementary Figure 2: N-palmitoylglycine is non-toxic to HER2/neu-positive SKBR3 breast cancer cells. HER2/neu-positive SKBR3 and HER2-normal MCF7 breast cancer cells were treated with palmitate, N-palmitoylglycine or vehicle for 24 and 48 hours (++ = 250 µM, + = 100 µM). Cells were fixed and neutral lipids were stained with BODIPY 493/503. Nuclei were stained with Hoechst 33342. BODIPY fluorescence and nuclei were imaged and quantified using the INCell Analyzer 2200 and INCell Investigator software. Fluorescence intensity per cell is proportional to the neutral lipid content in the cell. Statistical analysis was carried out in Graphpad Prism. Data are presented mean ± SD. * = $p \leq 0.05$, ** = $p \leq 0.01$, *** = $p \leq 0.001$, one-way ANOVA with Bonferroni post-test, n = 3.

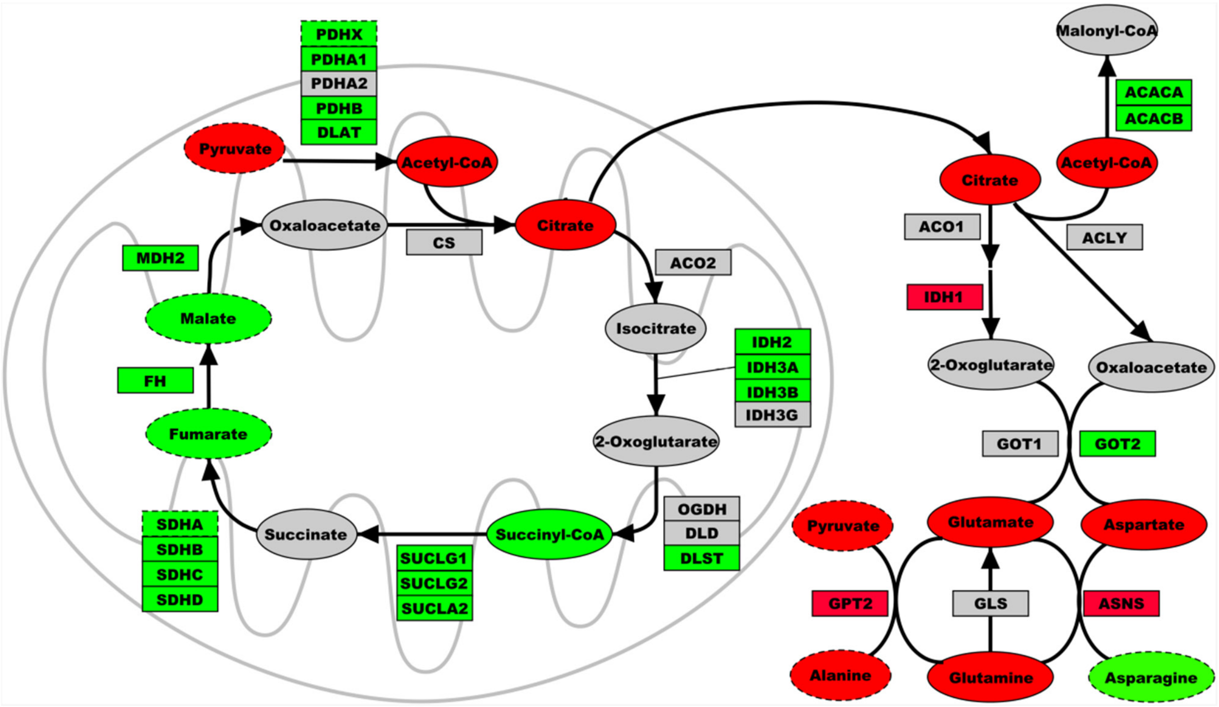

Supplementary Figure 3: Network-overlay of metabolomic and transcriptomic data indicates alterations in the TCA cycle. Detailed section of a network-overlay of metabolomic and transcriptomic data of SKBR3 cells after 24 h treatment with 250 µM palmitate. TCA cycle intermediates and enzyme mRNA levels change with palmitate treatment. Networks were created in Metscape, detailed section were ported into PathVisio and edited for clarity. Oval nodes indicate metabolites, boxes indicate genes. Red fill color denotes upregulation, green downregulation compared to control conditions. Grey nodes are part of the network but were not identified or did not change in abundance/expression. Dashed outline of a node indicates $p \leq 0.1$, whereas solid outlines indicate $p \leq 0.05$.

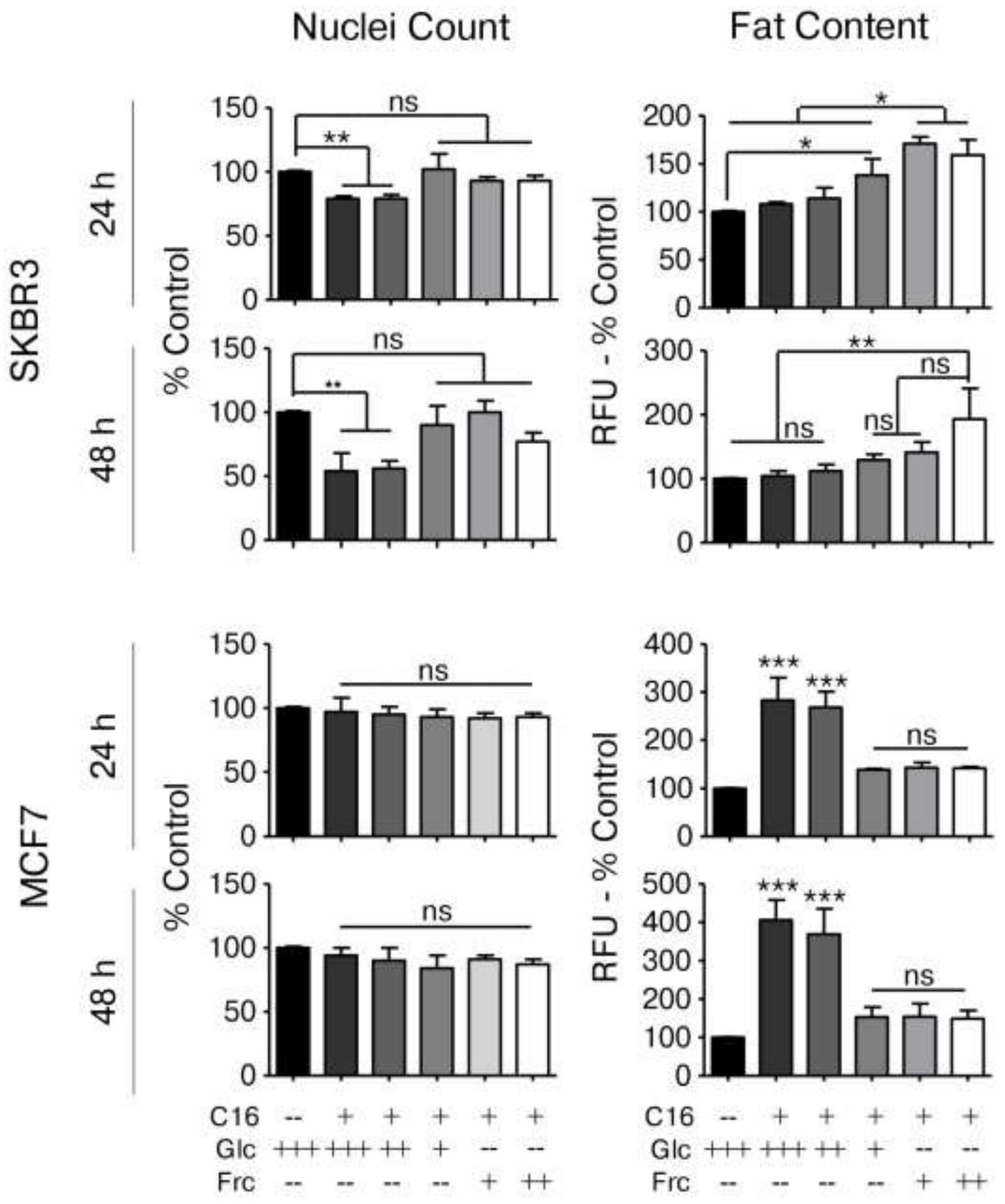

Supplementary Figure 4: Glucose metabolism mediates the lipotoxic effects of palmitate in HER2/neu-positive SKBR3 breast cancer cells. HER2/neu-positive SKBR3 and HER2-normal MCF7 breast cancer cells were treated with 250 µM palmitate or vehicle for 24 and 48 hours in growth media with decreasing glucose concentrations or fructose instead of glucose (+++ = 25 mM, ++ = 5 mM, + = 5 µM). Cells were fixed and neutral lipids were stained with BODIPY 493/503. Nuclei were stained with Hoechst 33342. BODIPY fluorescence and nuclei were imaged and quantified using the INCell Analyzer 2200 and INCell Investigator software. Fluorescence intensity per cell is proportional to the neutral lipid content in the cell. Statistical analysis was carried out in Graphpad Prism. Data are presented mean ± SD. * = $p \leq 0.05$, ** = $p \leq 0.01$, *** = $p \leq 0.001$, one-way ANOVA with Bonferroni post-test, n = 3.
