# Supplementary material for: Global metabolite profiling analysis of lipotoxicity in HER2/neu-positive breast cancer cells
Source: Oncotarget. 2018 Jun 5;9(43):27133–50. doi: 10.18632/oncotarget.25500 (PMC6007458; doi:10.18632/oncotarget.25500)
Supplement: Supplementary file 2 [file oncotarget-09-27133-s002.docx]

**Supplementary Table 1: Metabolite concentration table used as input for the bioinformatics resource at metaboanalyst.ca**

| Sample | **VEH_24_1** | **VEH_24_2** | **VEH_24_3** | **VEH_24_4** | **VEH_24_5** | **C16_24_1** | **C16_24_2** | **C16_24_3** | **C16_24_4** | **C16_24_5** |  |
| --- | --- | --- | --- | --- | --- | --- | --- | --- | --- | --- | --- |
| Label | ctrl | ctrl | ctrl | ctrl | ctrl | C16 | C16 | C16 | C16 | C16 | p.value |
| HMDB00222 | 0.6496 | 0.5861 | 0.6621 | 0.8863 | 0.6138 | 3.9629 | 4.704 | 4.4751 | 4.5827 | 4.535 | 3.579E-09 |
| HMDB00791 | 1.2847 | 1.0622 | 1.3069 | 1.3494 | 1.3548 | 0.5191 | 0.5222 | 0.5683 | 0.6107 | 0.4679 | 1.6597E-06 |
| HMDB00125 | 0.9908 | 0.9694 | 0.909 | 1.0673 | 0.9594 | 1.5804 | 1.8149 | 1.6453 | 1.5393 | 1.6005 | 2.0333E-06 |
| HMDB00929 | 0.9717 | 1.0007 | 0.846 | 0.9645 | 0.9386 | 1.4194 | 1.4769 | 1.4841 | 1.4473 | 1.2635 | 9.7335E-06 |
| HMDB00089 | 0.8146 | 0.7691 | 0.8621 | 0.9874 | 0.9049 | 1.5733 | 1.7169 | 1.5539 | 1.3649 | 1.4885 | 9.9403E-06 |
| HMDB00736 | 1.0126 | 0.7056 | 0.792 | 0.8654 | 0.8335 | 1.4205 | 1.3642 | 1.4273 | 1.418 | 1.2907 | 1.1816E-05 |
| HMDB60256 | 1.4762 | 1.7688 | 1.3775 | 1.5329 | 1.2647 | 0.6709 | 0.6674 | 0.7439 | 0.6146 | 0.5756 | 1.4592E-05 |
| HMDB00211 | 0.8623 | 0.9867 | 1.0057 | 1.0788 | 1.0213 | 0.5777 | 0.645 | 0.6378 | 0.6741 | 0.6175 | 1.5444E-05 |
| HMDB00210 | 1.0912 | 1.0501 | 1.0102 | 1.0443 | 1.069 | 1.3938 | 1.5411 | 1.4272 | 1.3534 | 1.3361 | 1.5502E-05 |
| HMDB01851 | 1.6941 | 2.024 | 1.5275 | 1.8788 | 1.5799 | 0.8627 | 0.9726 | 0.8395 | 0.804 | 0.7034 | 2.1683E-05 |
| HMDB02250 | 1.1175 | 0.9883 | 1.0323 | 1.2203 | 0.9285 | 0.5679 | 0.588 | 0.5935 | 0.635 | 0.5103 | 2.3694E-05 |
| HMDB00482 | 0.6436 | 0.7664 | 0.4578 | 0.6195 | 0.6309 | 1.2084 | 1.2325 | 1.4439 | 1.1282 | 1.1948 | 2.7976E-05 |
| HMDB03337 | 0.9504 | 0.8 | 0.7809 | 0.954 | 0.8194 | 1.3169 | 1.5621 | 1.2972 | 1.5097 | 1.591 | 3.6965E-05 |
| HMDB00705 | 1.3779 | 1.2692 | 1.498 | 1.5997 | 1.5814 | 0.7904 | 0.5756 | 0.8871 | 0.9043 | 0.6449 | 5.3033E-05 |
| HMDB01416 | 0.6921 | 0.5296 | 0.723 | 0.662 | 0.766 | 1.385 | 1.4776 | 2.0626 | 1.7669 | 1.9468 | 5.7262E-05 |
| HMDB01564 | 0.7057 | 0.6609 | 0.6594 | 0.7532 | 0.6761 | 1.1387 | 1.4322 | 1.0599 | 1.3146 | 1.1503 | 0.00006475 |
| HMDB00158 | 0.8583 | 0.8593 | 0.9631 | 1.0299 | 0.9002 | 1.2252 | 1.2433 | 1.2575 | 1.3123 | 1.1387 | 9.1257E-05 |
| HMDB00172 | 0.8413 | 0.9966 | 0.9633 | 1.0034 | 0.9687 | 1.2062 | 1.2099 | 1.3769 | 1.3587 | 1.3539 | 9.3309E-05 |
| HMDB01396 | 1.0975 | 1.052 | 1.0059 | 1.0289 | 1.0099 | 0.686 | 0.6864 | 0.8218 | 0.8627 | 0.7046 | 0.00011447 |
| HMDB03466 | 0.9573 | 1.0709 | 0.9594 | 1.0942 | 0.996 | 0.7549 | 0.8147 | 0.6679 | 0.7165 | 0.8132 | 0.00018148 |
| HMDB00071 | 0.9379 | 1.2703 | 1.156 | 0.9924 | 1.0504 | 0.4886 | 0.4986 | 0.76 | 0.6101 | 0.5002 | 0.00019844 |
| HMDB00687 | 0.6897 | 0.9648 | 0.9248 | 0.9753 | 0.953 | 1.2368 | 1.2208 | 1.3545 | 1.2975 | 1.2867 | 0.00020011 |
| HMDB00064 | 1.2871 | 1.337 | 1.0401 | 1.1964 | 1.1528 | 0.7915 | 0.9024 | 0.7102 | 0.811 | 0.8537 | 0.00021846 |
| HMDB00271 | 1.116 | 1.2555 | 1.0174 | 1.1392 | 1.2513 | 0.8233 | 0.8435 | 0.8839 | 0.8812 | 0.7623 | 0.00022311 |
| HMDB00078 | 0.8998 | 1.0627 | 0.8226 | 1.0099 | 0.9958 | 1.8333 | 2.1866 | 1.528 | 1.496 | 1.7929 | 0.00028003 |
| HMDB01173 | 0.7073 | 0.6878 | 0.7762 | 0.8991 | 0.8584 | 1.2023 | 1.2508 | 1.6465 | 1.6839 | 1.3269 | 0.00039881 |
| HMDB00640 | 0.8667 | 1.3995 | 1.4824 | 0.9822 | 1.1856 | 0.4185 | 0.4185 | 0.6225 | 0.4185 | 0.4185 | 0.00039899 |
| HMDB00098 | 1.1256 | 1.8977 | 1.2311 | 1.8734 | 1.4144 | 0.6942 | 0.606 | 0.4955 | 0.4955 | 0.4955 | 0.00043527 |
| HMDB00224 | 0.7824 | 0.9848 | 0.6752 | 0.8992 | 1.0036 | 0.3623 | 0.5105 | 0.4726 | 0.4778 | 0.5468 | 0.0004631 |
| HMDB00848 | 0.9883 | 0.9634 | 0.8677 | 1.1363 | 0.7777 | 1.4847 | 2.4566 | 2.7118 | 2.1752 | 2.0379 | 0.00046971 |
| HMDB00641 | 1.1319 | 1.2512 | 0.8764 | 0.9596 | 1.2034 | 1.5394 | 1.7979 | 1.6897 | 1.9043 | 1.4758 | 0.00052205 |
| HMDB03229 | 0.7592 | 0.8202 | 0.5264 | 0.4246 | 0.7168 | 1.7442 | 1.4553 | 2.4263 | 2.3589 | 1.4612 | 0.00056797 |
| HMDB11738 | 1.3683 | 1.1004 | 0.8191 | 1.1904 | 1.0091 | 1.7986 | 2.5256 | 1.7857 | 1.9603 | 1.8744 | 0.00065257 |
| HMDB05765 | 1.3838 | 1.1346 | 1.5475 | 1.8029 | 1.3033 | 0.5773 | 0.6883 | 0.8008 | 0.9496 | 0.7202 | 0.00072078 |
| HMDB00095 | 1.0266 | 0.7268 | 0.8297 | 0.8792 | 0.8443 | 1.2754 | 1.4622 | 1.4398 | 1.7168 | 1.158 | 0.00086225 |
| HMDB00755 | 0.9258 | 0.8819 | 0.7763 | 0.8286 | 0.8017 | 1.0841 | 1.21 | 1.072 | 1.0704 | 0.9702 | 0.00093787 |
| HMDB13716 | 0.9691 | 1.0164 | 0.8103 | 0.9369 | 0.9521 | 1.2756 | 1.4565 | 1.3496 | 1.3259 | 1.0791 | 0.00095144 |
| HMDB00045 | 1.1036 | 0.9589 | 1.0094 | 1.3044 | 0.9906 | 1.5493 | 2.0187 | 1.4927 | 1.5336 | 1.6189 | 0.0011064 |
| HMDB00671 | 0.7048 | 0.7116 | 0.5289 | 0.7027 | 0.5674 | 1.3066 | 1.3101 | 0.9851 | 1.1934 | 0.8503 | 0.0012112 |
| HMDB01373 | 0.966 | 1.143 | 1.1661 | 0.9177 | 1.0833 | 2.0929 | 1.6213 | 2.7204 | 2.3103 | 1.6561 | 0.0013064 |
| HMDB00267 | 0.8876 | 1.0196 | 0.956 | 1.0099 | 0.961 | 1.0401 | 1.1361 | 1.2002 | 1.0618 | 0.9795 | 0.0013067 |
| HMDB11741 | 0.8121 | 0.691 | 0.6127 | 0.6127 | 0.6127 | 1.5648 | 1.1106 | 1.653 | 0.9484 | 1.4179 | 0.0013611 |
| HMDB11737 | 0.9872 | 0.6566 | 0.8903 | 1.0128 | 0.9688 | 1.2173 | 1.6283 | 1.5252 | 1.7336 | 1.2523 | 0.0015555 |
| HMDB01015 | 1.0149 | 1.001 | 0.9136 | 1.037 | 0.8688 | 1.4113 | 1.4987 | 1.2172 | 1.1916 | 1.1813 | 0.0017871 |
| HMDB00159 | 0.9984 | 0.8782 | 0.9958 | 1.0653 | 0.9897 | 1.1506 | 1.2424 | 1.2961 | 1.2994 | 1.0876 | 0.0021031 |
| HMDB00191 | 1.0425 | 1.0233 | 1.0556 | 1.1879 | 1.2633 | 1.2413 | 1.4469 | 1.4753 | 1.4103 | 1.3824 | 0.0021598 |
| HMDB00935 | 1.0381 | 1.0116 | 1.0478 | 1.1328 | 0.9686 | 1.3083 | 1.4281 | 1.4324 | 1.3562 | 1.111 | 0.0021648 |
| HMDB01586 | 2.4848 | 2.2192 | 1.8122 | 1.4181 | 1.2109 | 0.7009 | 0.984 | 0.6235 | 0.6543 | 0.7116 | 0.002176 |
| HMDB01257 | 0.9498 | 0.826 | 1.0717 | 0.9465 | 0.8074 | 1.1596 | 1.1699 | 1.5496 | 1.2544 | 1.3766 | 0.0023972 |
| HMDB00220 | 0.6678 | 0.9366 | 0.6069 | 0.4992 | 0.7544 | 1.516 | 1.4177 | 2.4121 | 1.9834 | 1.1752 | 0.0025217 |
| HMDB00594 | 0.5597 | 0.8162 | 0.7578 | 0.9654 | 1.1437 | 1.1683 | 1.4766 | 1.5534 | 1.7804 | 1.3138 | 0.0027925 |
| HMDB41623 | 0.9478 | 1.0453 | 0.9149 | 1.1501 | 0.9361 | 1.5419 | 1.3014 | 1.3727 | 1.1018 | 1.4168 | 0.0034509 |
| HMDB05065 | 1.0054 | 0.9118 | 0.8715 | 1.1281 | 0.7925 | 0.7463 | 0.7446 | 0.5179 | 0.6198 | 0.5645 | 0.0035029 |
| HMDB00214 | 0.767 | 0.9357 | 1.0396 | 1.1246 | 0.9973 | 1.1913 | 1.5741 | 1.194 | 1.4785 | 1.5455 | 0.003515 |
| HMDB00883 | 0.829 | 0.8904 | 1.0874 | 1.0322 | 1.0191 | 1.3625 | 1.291 | 1.2048 | 1.5985 | 1.1861 | 0.0038148 |
| HMDB01517 | 1.4762 | 0.9767 | 1.4875 | 1.2204 | 2.4314 | 0.5103 | 0.5103 | 0.5103 | 0.5563 | 0.5648 | 0.0039618 |
| HMDB00148 | 1.1351 | 1.0549 | 0.9355 | 0.98 | 1.0036 | 1.2288 | 1.2311 | 1.2947 | 1.3439 | 1.0852 | 0.0046739 |
| HMDB00067 | 0.822 | 0.9842 | 0.9656 | 1.0267 | 0.9325 | 1.05 | 1.1417 | 1.0642 | 1.1179 | 1.0974 | 0.0048887 |
| HMDB00167 | 0.9967 | 0.9418 | 0.9165 | 0.9473 | 1.0033 | 1.1032 | 1.2932 | 1.3014 | 1.387 | 1.0311 | 0.0051817 |
| HMDB00131 | 0.8537 | 0.9662 | 0.8991 | 0.9696 | 0.9338 | 1.1172 | 1.1489 | 1.2059 | 1.1908 | 0.9378 | 0.0060517 |
| HMDB11635 | 0.6709 | 0.9167 | 0.8612 | 0.8992 | 0.8507 | 1.0993 | 1.1205 | 1.5512 | 1.521 | 1.0654 | 0.0061401 |
| HMDB29108 | 0.8634 | 0.94 | 0.7154 | 0.4605 | 1.0266 | 1.6542 | 1.5184 | 1.0563 | 1.1036 | 1.4795 | 0.0068027 |
| HMDB02271 | 1.1744 | 0.9278 | 0.8424 | 0.8684 | 0.9548 | 0.6923 | 0.7941 | 0.7849 | 0.5706 | 0.6175 | 0.0074587 |
| HMDB00122 | 1.008 | 1.118 | 1.4788 | 1.8986 | 1.1463 | 0.7732 | 0.7529 | 0.6603 | 0.6822 | 0.9004 | 0.0089257 |
| HMDB00251 | 0.9802 | 0.9081 | 1.3769 | 1.0175 | 1.0888 | 0.4899 | 0.4015 | 0.8762 | 0.8757 | 0.4861 | 0.009161 |
| HMDB28850 | 0.9626 | 1.0058 | 1.3818 | 1.0233 | 1.3332 | 0.7667 | 0.8615 | 0.7206 | 0.9073 | 0.8274 | 0.0091821 |
| HMDB00688 | 0.964 | 1.1067 | 1.0804 | 1.1133 | 1.0493 | 1.4502 | 1.4461 | 1.5058 | 1.387 | 1.036 | 0.0092169 |
| HMDB28997 | 1.5187 | 1.2112 | 1.5522 | 1.0961 | 0.888 | 0.9924 | 0.8183 | 0.5296 | 0.7637 | 0.5296 | 0.0092692 |
| HMDB01206 | 0.8102 | 0.8825 | 1.0047 | 1.0284 | 1.1075 | 1.0363 | 1.2355 | 1.1849 | 1.2924 | 1.3046 | 0.009538 |
| HMDB00272 | 0.7996 | 0.9391 | 0.8144 | 0.9261 | 0.9306 | 0.9674 | 1.6979 | 1.4805 | 2.3352 | 1.6395 | 0.010096 |
| HMDB00187 | 0.8047 | 0.9037 | 0.9805 | 1.0411 | 1.0584 | 1.1099 | 1.1858 | 1.166 | 1.1871 | 1.0411 | 0.010813 |
| HMDB01022 | 1.6071 | 1.2309 | 2.2466 | 2.0227 | 1.4105 | 1 | 0.691 | 0.5119 | 1.5122 | 0.5119 | 0.01244 |
| HMDB00221 | 0.9121 | 1.0994 | 1.0376 | 1.0529 | 1.0942 | 1.1662 | 1.2383 | 1.2973 | 1.1162 | 1.14 | 0.012613 |
| HMDB01539 | 0.7 | 0.6884 | 0.7153 | 0.9743 | 0.9169 | 0.9934 | 1.2856 | 1.3576 | 1.7683 | 1.017 | 0.013224 |
| HMDB00085 | 1.1898 | 1.4064 | 1.1617 | 0.794 | 1.1882 | 0.6417 | 0.6621 | 0.9686 | 0.8369 | 0.335 | 0.013524 |
| HMDB28999 | 1.012 | 0.6971 | 1.0824 | 0.9687 | 0.8529 | 0.6971 | 0.6971 | 0.6971 | 0.7702 | 0.6971 | 0.015825 |
| HMDB00174 | 0.9974 | 1.0778 | 0.9813 | 0.5711 | 1.0026 | 0.9753 | 2.2324 | 1.5243 | 1.466 | 1.7997 | 0.017412 |
| HMDB00177 | 0.8557 | 0.8705 | 0.9428 | 0.8992 | 1.0229 | 1.0052 | 1.0382 | 1.2529 | 1.1779 | 0.9989 | 0.017806 |
| HMDB00143 | 1.6247 | 1.9377 | 1.1118 | 0.7895 | 1.266 | 0.561 | 0.6456 | 0.9933 | 0.7424 | 0.7254 | 0.020489 |
| HMDB28757 | 0.9314 | 1.3808 | 1.1728 | 0.9966 | 1.2169 | 0.9634 | 0.9967 | 0.6648 | 0.8654 | 0.7923 | 0.02235 |
| HMDB28988 | 1.9968 | 1.0238 | 2.2763 | 1.4703 | 1.0608 | 0.7478 | 0.7676 | 0.7586 | 1.1566 | 0.6794 | 0.022586 |
| HMDB29109 | 1.7501 | 1.4653 | 2.383 | 1.7933 | 1.1491 | 1.0293 | 1.4503 | 1.0703 | 0.9781 | 0.6802 | 0.023433 |
| HMDB02917 | 1.2904 | 2.0376 | 1.7732 | 0.659 | 2.1956 | 0.898 | 0.549 | 1.095 | 0.6516 | 0.6905 | 0.024887 |
| HMDB00273 | 0.9163 | 1.3925 | 0.9231 | 0.6237 | 1.0778 | 0.4003 | 0.3713 | 0.5928 | 0.9282 | 0.3713 | 0.024917 |
| HMDB00229 | 0.7111 | 0.6107 | 0.8145 | 0.8071 | 0.9577 | 1.2485 | 0.8684 | 1.394 | 1.3256 | 0.8227 | 0.029195 |
| HMDB01423 | 1.1111 | 0.9806 | 1.0049 | 1.103 | 0.9951 | 1.1213 | 1.364 | 1.1052 | 1.2364 | 1.1156 | 0.031118 |
| HMDB00766 | 1.0491 | 0.9951 | 0.9298 | 0.958 | 1.0689 | 0.7211 | 0.9467 | 0.9172 | 0.9408 | 0.7575 | 0.031612 |
| HMDB00162 | 1.2085 | 0.9427 | 0.9087 | 1 | 0.9538 | 1.1978 | 1.2665 | 1.2869 | 1.672 | 1.0688 | 0.032145 |
| HMDB00965 | 0.9713 | 0.8015 | 1.6202 | 1.5438 | 1.0771 | 0.7107 | 0.7435 | 0.7324 | 0.8944 | 0.8022 | 0.032285 |
| HMDB00157 | 0.8933 | 0.9595 | 0.9503 | 0.9815 | 1.0213 | 1.0285 | 1.0393 | 1.357 | 1.2755 | 1.0411 | 0.033262 |
| HMDB00124 | 0.9002 | 1.1674 | 0.6946 | 1.0387 | 1.0714 | 0.8007 | 0.756 | 0.6949 | 0.5313 | 0.8421 | 0.034889 |
| HMDB00767 | 0.6004 | 0.9763 | 0.9233 | 0.959 | 1.0959 | 1.2915 | 1.211 | 1.6508 | 1.0374 | 1.0761 | 0.037236 |
| HMDB00508 | 1.21 | 1.254 | 1.2462 | 1.1259 | 1.1648 | 1.0154 | 1.2883 | 0.9252 | 0.7781 | 0.859 | 0.037596 |
| HMDB29136 | 1.679 | 0.8933 | 1.7476 | 1.2984 | 0.993 | 0.7157 | 1.1162 | 0.5295 | 0.9795 | 0.7744 | 0.038229 |
| HMDB00186 | 0.9372 | 0.7462 | 0.9842 | 0.8901 | 0.997 | 0.9709 | 0.9842 | 1.1347 | 1.1234 | 1.0342 | 0.040512 |
| HMDB00325 | 0.7461 | 0.8642 | 1.0292 | 1.3748 | 0.7719 | 1.2376 | 1.9013 | 1.1959 | 1.1657 | 2.1342 | 0.041241 |
| HMDB00650 | 1.0176 | 1.1921 | 0.993 | 1.0831 | 1.1126 | 0.9157 | 1.0448 | 1.007 | 0.9754 | 0.8266 | 0.042263 |
| HMDB00182 | 1.0628 | 0.7749 | 0.7582 | 0.7302 | 0.9404 | 1.0029 | 1.2006 | 1.2751 | 1.3669 | 0.8237 | 0.043357 |
| HMDB00056 | 0.9241 | 0.7131 | 1.5757 | 1.4617 | 1.0019 | 0.7371 | 0.6504 | 0.6793 | 0.8806 | 0.7585 | 0.048274 |
| HMDB01413 | 1.0266 | 1.088 | 0.6749 | 0.6331 | 1.0005 | 1.351 | 1.1668 | 1.0921 | 1.478 | 0.9184 | 0.049339 |
| HMDB00019 | 0.4391 | 0.3194 | 0.3194 | 0.3194 | 0.3194 | 1.0918 | 1.1008 | 1.0834 | 0.3194 | 0.3194 | 0.050127 |
| HMDB02817 | 1.0247 | 0.9341 | 0.8194 | 0.7104 | 0.7686 | 0.8942 | 1.1421 | 1.2357 | 1.0297 | 0.9261 | 0.053712 |
| HMDB01401 | 0.9109 | 1.1952 | 0.6222 | 1.0548 | 1.1938 | 0.8403 | 0.7627 | 0.778 | 0.4958 | 0.7186 | 0.053792 |
| HMDB02075 | 1.1799 | 1 | 0.5397 | 0.9653 | 0.8052 | 1.3626 | 0.9017 | 1.9143 | 2.3811 | 1.1747 | 0.053863 |
| HMDB28848 | 1.3646 | 0.7757 | 1.2711 | 0.9925 | 0.9998 | 0.8545 | 0.6158 | 0.7519 | 1.0616 | 0.6158 | 0.056248 |
| HMDB01406 | 1.0531 | 1.0781 | 1.0664 | 0.9725 | 1.021 | 0.8931 | 0.9062 | 1.0487 | 1.0146 | 0.83 | 0.056435 |
| HMDB00213 | 0.839 | 1.0121 | 1.218 | 1.1951 | 1.2739 | 0.8903 | 0.8204 | 0.9779 | 0.9705 | 0.9443 | 0.060153 |
| HMDB02931 | 0.9307 | 0.988 | 1.0097 | 1.017 | 1.017 | 0.9529 | 0.8938 | 0.9439 | 0.9862 | 0.8135 | 0.061192 |
| HMDB00706 | 1.0904 | 1.9195 | 0.7209 | 0.7554 | 1.3743 | 0.7538 | 0.5786 | 0.7951 | 0.7465 | 0.5257 | 0.06302 |
| HMDB00696 | 0.9126 | 1.0551 | 0.8786 | 1.0235 | 1.0376 | 1.0299 | 1.2149 | 1.2603 | 1.2547 | 0.9441 | 0.063388 |
| HMDB01248 | 0.8464 | 0.8559 | 1.0196 | 1.0402 | 0.9874 | 0.9997 | 1.0148 | 1.2172 | 1.2058 | 1.0037 | 0.066351 |
| HMDB29137 | 1.4134 | 1.1729 | 1.4393 | 1.2066 | 0.7833 | 0.7694 | 1 | 0.7651 | 1.0985 | 0.952 | 0.066475 |
| HMDB03736 | 0.5935 | 0.4983 | 0.3522 | 0.7102 | 0.2776 | 0.919 | 1.2131 | 0.425 | 0.5869 | 1.0321 | 0.066875 |
| HMDB00806 | 1.1493 | 1.5426 | 1.0156 | 0.7894 | 1.2461 | 0.8123 | 0.6311 | 1.091 | 0.9545 | 0.6459 | 0.068848 |
| HMDB01414 | 0.9888 | 0.8363 | 1.2146 | 1.1897 | 0.8443 | 1.7581 | 1.8127 | 1.1284 | 1.0112 | 1.2688 | 0.071344 |
| HMDB00094 | 0.8017 | 0.5957 | 0.9679 | 1.6949 | 0.728 | 1.1906 | 2.4414 | 1.1126 | 1.3703 | 1.9571 | 0.074007 |
| HMDB00289 | 0.9782 | 0.6743 | 1.2618 | 1.1818 | 0.7983 | 0.784 | 0.7006 | 0.5567 | 0.7872 | 0.8389 | 0.078187 |
| HMDB00168 | 0.9506 | 1.2752 | 0.7893 | 0.9973 | 1.2808 | 0.7206 | 0.8295 | 0.9866 | 0.8749 | 0.8327 | 0.081044 |
| HMDB01227 | 1.1308 | 1.0433 | 1.033 | 1.1649 | 1.0024 | 0.9916 | 1.1018 | 0.9976 | 0.8035 | 0.885 | 0.08229 |
| HMDB00695 | 0.6561 | 0.5434 | 0.3404 | 0.8245 | 0.4666 | 0.9391 | 1.2977 | 0.6169 | 0.5616 | 0.9743 | 0.082523 |
| HMDB05066 | 1.0123 | 0.7829 | 0.9877 | 1.1611 | 0.8555 | 0.7224 | 0.7881 | 0.6728 | 0.8103 | 0.9784 | 0.083609 |
| HMDB01341 | 0.9801 | 1.0214 | 1.0779 | 1.1854 | 1.0054 | 1.1071 | 1.7326 | 1.265 | 1.0986 | 1.2623 | 0.084117 |
| HMDB00625 | 0.577 | 0.6727 | 0.8419 | 0.8924 | 1.0233 | 1.0945 | 0.9923 | 1.3193 | 0.7559 | 1.0302 | 0.084661 |
| HMDB00638 | 0.9723 | 1.2255 | 0.9535 | 0.8406 | 0.9776 | 0.7959 | 0.6961 | 0.9996 | 0.9367 | 0.6267 | 0.08872 |
| HMDB01893 | 0.8236 | 1.0107 | 0.8289 | 0.9725 | 0.3117 | 1.4454 | 1.0817 | 1.0761 | 0.9529 | 0.9013 | 0.091 |
| HMDB28995 | 0.7888 | 1.0264 | 1.2268 | 1.3518 | 1.0699 | 0.8057 | 0.9081 | 0.909 | 0.8543 | 1.0121 | 0.091096 |
| HMDB01262 | 0.3355 | 0.7794 | 1.0462 | 0.5506 | 0.8707 | 4.7693 | 1.2785 | 1.4405 | 1.7889 | 0.9538 | 0.095992 |
| HMDB28837 | 0.6897 | 0.8097 | 0.9924 | 1.104 | 0.9227 | 0.9563 | 1.1721 | 1.2234 | 0.9994 | 1.0006 | 0.099125 |
| HMDB11617 | 0.9681 | 1.5711 | 1.0606 | 0.634 | 1.3957 | 0.6536 | 0.3299 | 1.1673 | 0.9413 | 0.4097 | 0.099285 |

Metabolites with significantly altered concentrations in SKBR3 cells after 24hrs of treatment with cells after 24h treatment with 250 µM palmitate (C16_24 samples) or vehicle control (VEH_24 samples). Metabolites are listed by Human Metabolome Database identification number.
